# Genome-wide association study reveals SNP markers controlling drought tolerance and related agronomic traits in chickpea across multiple environments

**DOI:** 10.3389/fpls.2024.1260690

**Published:** 2024-03-08

**Authors:** Tawffiq Istanbuli, Ahmed E. Nassar, Mamdouh M. Abd El-Maksoud, Sawsan Tawkaz, Alsamman M. Alsamman, Aladdin Hamwieh

**Affiliations:** ^1^ Biotechnology Department, International Center for Agricultural Research in the Dry Areas (ICARDA), Terbol, Lebanon; ^2^ Biotechnology Department, International Center for Agricultural Research in the Dry Areas (ICARDA), Giza, Egypt; ^3^ Department of Genetics, Faculty of Agriculture, Mansoura University, Mansoura, Egypt; ^4^ Genome Mapping Department, Agricultural Genetic Engineering Research Institute (AGERI), Agricultural Research Center (ARC), Giza, Egypt

**Keywords:** chickpea, drought-tolerance, GWAS, morphological and yield traits, rain-fed, irrigated, diversity

## Abstract

Chickpea, renowned for its exceptional nutritional value, stands as a crucial crop, serving as a dietary staple in various parts of the world. However, its productivity faces a significant challenge in the form of drought stress. This challenge highlights the urgent need to find genetic markers linked to drought tolerance for effective breeding programs. The primary objective of this study is to identify genetic markers associated with drought tolerance to facilitate effective breeding programs. To address this, we cultivated 185 chickpea accessions in two distinct locations in Lebanon over a two-year period, subjecting them to both irrigated and rain-fed environments. We assessed 11 drought-linked traits, including morphology, growth, yield, and tolerance score. SNP genotyping revealed 1344 variable SNP markers distributed across the chickpea genome. Genetic diversity across populations originating from diverse geographic locations was unveiled by the PCA, clustering, and structure analysis indicating that these genotypes have descend from five or four distinct ancestors. A genome-wide association study (GWAS) revealed several marker trait associations (MTAs) associated with the traits evaluated. Within the rainfed conditions, 11 significant markers were identified, each associated with distinct chickpea traits. Another set of 11 markers exhibited associations in both rainfed and irrigated environments, reflecting shared genetic determinants across these conditions for the same trait. The analysis of linkage disequilibrium (LD) highlighted two genomic regions with notably strong LD, suggesting significant interconnections among several investigated traits. This was further investigated by the correlation between major markers associated with these traits. Gene annotation of the identified markers has unveiled insights into 28 potential genes that play a role in influencing various chickpea drought-linked traits. These traits encompass crucial aspects such as blooming organ development, plant growth, seed weight, starch metabolism, drought regulation, and height index. Among the identified genes are *CPN60-2*, *hsp70*, *GDSL(GELP)*, *AHL16*, *NAT3*, *FAB1B*, *bZIP*, and *GL21*. These genes collectively contribute to the multifaceted response of chickpea plants to drought stress. Our identified genetic factors exert their influence in both irrigated and rainfed environments, emphasizing their importance in shaping chickpea characteristics.

## 1 Introduction

The chickpea (*Cicer arietinum L.*) is a grain legume and one of the seven Neolithic founding crops of the Near Eastern Fertile Crescent. It is commonly known as garbanzo beans or Bengal gram (Ujinwal et al., 2019). Chickpea is grown in over fifty countries, including North Africa, the Middle East, southern Europe, the Americas, and Australia, making them the third most important legume crop in the world. It is often referred to as “poor man’s meat” because of its high nutritional value. Chickpea is rich in starch, which is the main component of the carbohydrate fraction, fat content, and protein. The fat content ranges from 3.10%–5.67%, and protein ranges from 20% to 25%. India is the leading producer of chickpeas, with an annual production of 11.5 million tons (Merga and Haji, 2019; Grasso et al., 2022). The cultivated Cicer genus is believed to be the domesticated one, which evolved from the wild species, *Cicer reticulatum*. Chickpeas are classified as “desi” or “kabuli” based on their color, size, and seed. Desi species are found primarily in semiarid regions and are distinguished by small, dark brown, wrinkled seeds. Kabuli species are distinguished by their sizable seed, smooth surface, and growth at various temperatures (Wrigley et al., 2015; Maya and Maphosa, 2020).

Climate change is considered one of the most severe threats to the earth’s ecosystems. Anthropogenic activities, particularly greenhouse gas (GHG) emissions, have led to a temperature increase of 0.9°C since the 18th century, with projections indicating that this ratio could reach 1.5°C or higher by 2050 (Arora, 2019; Srivastav et al., 2019). This increase leads to floods, irregular precipitation patterns, heat waves, and droughts, causing global economic losses of 225 billion in 2018 (Arora, 2019). Drought and heat stress cause up to 50% of crop yield losses, affecting chickpea as a rotation crop that experiences moisture stress towards the end of the growing season (Devasirvatham and Tan, 2018; Singh et al., 2021). For every 1°C above the optimum temperature, there is a 10%-15% reduction in overall crop yield and a decrease of 53 kg/ha in chickpea yields (Dubey et al., 2011; Upadhyaya et al., 2011). Drought is responsible for 40%-45% of global chickpea yield losses, with a 30%-100% production loss in the WANA region during the spring drought (Devasirvatham and Tan, 2018; Rani et al., 2020). Earlier studies have identified indirect techniques to improve chickpea drought resistance, but a heritable and more effective genetic approach has not been documented. Identifying and utilizing genes involved in drought and heat tolerance can aid in developing climate-resilient chickpea varieties through breeding programs. This approach can potentially mitigate the negative impacts of climate change on chickpea production (Ramamoorthy et al., 2016; Mallikarjuna et al., 2022).

The current study represents a step forward in improving chickpea adaptation to drought by utilizing GWAS analysis to perform SNP (Single Nucleotide Polymorphisms) annotation and identify drought-related genes. GWAS is a valuable tool for mapping physiologically and economically traits in a wide range of genetic populations (Nassar et al., 2018; Alsamman et al., 2024). It has also been used in chickpeas and other legumes, such as lentils, soybeans, and peas, to investigate genetic diversity and identify marker-trait relationships for agronomic, biotic, abiotic, and nutritional variables (Srungarapu et al., 2022). Recent advancements in next-generation sequencing technologies, as well as the availability of applicable bioinformatics tools, such as GWAS, have aided in the identification of disease-associated SNPs (Roorkiwal et al., 2013). Genotyping technologies are being developed to leverage SNPs found in eukaryotic genomes. It has been reported that in birds like chickens, there is an average diversity of about one SNP every 200 bases for almost every possible comparison between two lines. Meanwhile, SNPs are common in plants, though their frequency appears to vary from species to species (Liu et al., 2023; Rizk, 2023). For example, in soybeans, one SNP was discovered every 270 bp, whereas in maize, it was found every 60 bp, and in Pisum, the change was noticed by one SNP every 20 bases in intronic regions (Ching et al., 2002; Jing et al., 2007). Even bi-allelic SNP markers could be customized for genetic mapping, marker-assisted selection (MAS), genomic selection (GS), structure, and genetic diversity (Deulvot et al., 2010; Gujaria et al., 2011).

Given the ongoing environmental changes, it is crucial to invest extensive efforts in developing new resilient and improved chickpea genotypes. The primary objective of this study is to tackle the challenge of drought stress and its impact on chickpea productivity. This is achieved by identifying genetic selection markers for drought tolerance to facilitate effective breeding programs. The study conducted a comprehensive analysis of 185 diverse chickpea genotypes in two environments across two years. The genotypes were subjected to different water treatments, including rainfed and irrigated conditions, while assessing various traits related to morphology, plant growth, yield, and drought tolerance. Through diversity analysis, the genetic diversity among the genotypes was explored. Genome-wide association mapping was employed to identify markers associated with the evaluated traits. Subsequent analyses were conducted to investigate the functions and potential interactions of these markers.

## 2 Methodology

### 2.1 Plant material and experimental procedure

Employing the Focused Identification of Germplasm Strategy (FIGS) for improving biological nitrogen fixation (BNF) screening, 204 chickpea accessions were acquired from ICARDA’s Genetic Resource Section (GRS) to uncover genes affecting chickpea characteristics (**Supplementary Tables 1**, and **2**). The experiment was conducted in two regions of Lebanon: Terbol, which is situated at 33°49’N and 35°59’E, at an altitude of 890 meters above sea level, and Kfardan, located at 30°01’N and 36°03’E, at an altitude of 1080 meters above sea level (Istanbuli et al., 2022). The plants were grown in an alpha lattice design with 35 cm between rows and 2.5 m row length (25 plants per row) in two replications over two seasons, from 2016 to 2017 (8 environments). The experiment included irrigated and rainfed treatments, with Rainfed Terbol (2016), Rainfed-Kfardan (2016), Rainfed-Terbol (2017), Rainfed-Kfardan (2017), Irrigated-Terbol (2016), Irrigated-Kfardan (2016), Irrigated-Terbol (2017), and Irrigated-Kfardan (2017). Both regions were treated with N:P:K (15:15:15), insecticide (Chlorpyrifos 48% EC), and fungicide (Chlorothalonil 37.5%).

### 2.2 Evaluated phenotypic traits for chickpea genotypes

#### 2.2.1 Traits evaluation

The chickpea genotypes were evaluated based on various morphological features, including nodule dry weight (NDW), nodule biomass (NB), nodule fresh weight (NFW), plant height (PLH), Height Index (HI), days to maturity (DM), and days to 50% flowering (DFL). To obtain data for NB, NDW, and NFW, three plants were chosen randomly from each water treatment, replication, and location throughout the flowering stage. DM and DFL were evaluated based on the soil’s capacity for germination after irrigation. To compute NB, the average volume of three plants was taken in m^3^, while NFW was evaluated after removing root nodules. NDW was determined by drying plants for two days at 48°C. During the two seasons (2016-2017), grain yield (GY) was assessed by collecting three plants from each treatment and location, and determining their weight after cleaning. Biological yield (BY) was determined by measuring the average dry weight of three shoots. In addition, the 100 seed weight (100SW) trait was evaluated using seed sub-samples. For screening chickpea drought tolerance scores, ICARDA created a scale from one to nine (Sabaghpour et al., 2006), with one being free of infection and each higher score indicating less resistance until nine is definitively dead (1 = free, 2 = greatly tolerant, 3 = tolerant, 4 = mildly tolerant, 5 = intermediately tolerant, 6 = modestly vulnerable, 7 = vulnerable, 8 = increased susceptibility, and 9 = death). The field data was analyzed using the GenStat program version 19, and REML meta-analysis was used to provide a comprehensive evaluation of the field experiments. Each environment was systematically examined, with genotype treated as a random variable, resulting in the identification of 87 trials that included 11 traits across 8 different environments. Variance components attributable to genotypes $\left({\;}_{ɡ}^{\;\sigma 2}\right)$ and errors $\left({\;}_{e}^{\;\sigma 2}\right)$ , along with their corresponding standard errors, were meticulously determined. The estimation of heritability was an integral facet of the analysis. The fixed factor of the year and the random interaction between genotypes (G) and environments (E) were judiciously considered. The Wald statistic was used to assess the fixed significance of the year. Predicted means from two replicates were employed to represent the resulting dataset, along with their standard error of the mean (SEM). Istanbuli et al. (2022) published thorough information about the experimental design and statistical analysis, employed in analyzing field data. In order to validate the previous analysis, we used ANOVA (Analysis of Variance) in this study to the interaction between the studied agronomic traits.

### 2.3 DNA isolation and SNP genotyping

DNA was extracted from 4- to 6-week-old seedlings according to the CTAB (cetyltrimethylammonium bromide) procedure (Li et al., 2020). The fresh leaf sample from seedlings was finely ground into a paste, which was then placed in a 2 mL Eppendorf tube containing 2X CTAB buffer consisting of 2% CTAB, 0.1 M Tris-HCl (pH 8.0), 1.4 M NaCl, and 20 mM ethylenediaminetetraacetic acid (EDTA). The suspension was then incubated for 15 minutes at 55°C in a recirculating water bath. Following incubation, 1 mL of 24:1 chloroform:isoamyl alcohol was added and the tube was shaken thoroughly. The tubes were then spun in the centrifuge at 4,500 rpm for 5 minutes, and the upper aqueous phase containing DNA was transferred to a microfuge tube. The DNA was washed twice with 0.54 volumes of 70% ethanol and 200 mM sodium acetate for 20 minutes. The DNA was then spun into a pellet at 13,000 rpm for 1 minute, and the supernatant was removed. The pellet was then dried for 20 minutes. To conduct a test gel, the DNA was re-suspended in 200 *µ*l of TE buffer for 1 hour at 55°C or overnight in the refrigerator.

**Figure 1:**
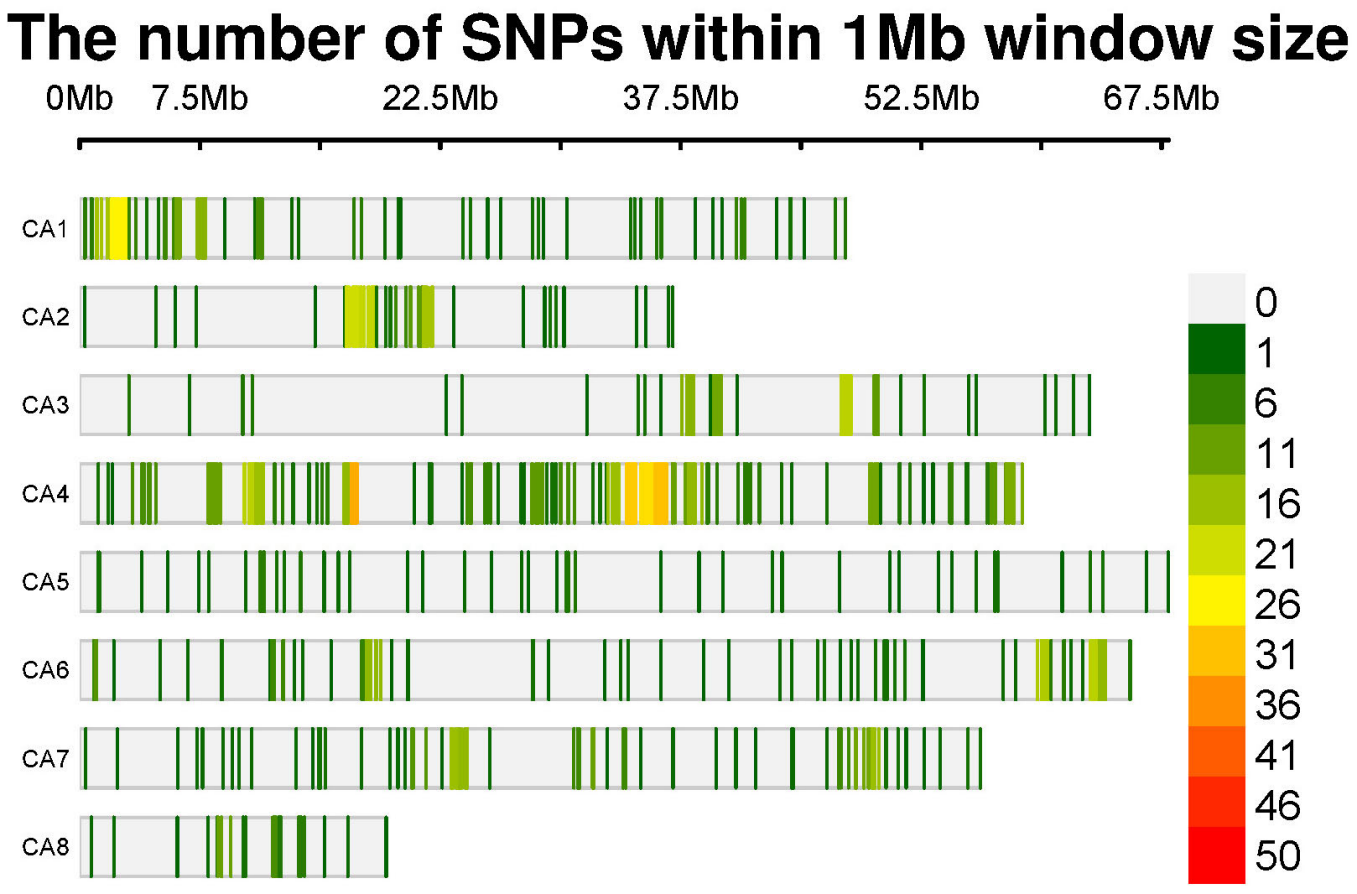
SNP-density plot of 1344 SNPs across chickpea chromosomes within a 1 MB window size.

For the sequencing of chickpea samples, we employed Diversity Arrays Technology (DArT®), a technology commonly used for SNP genotyping. A total of 204 DNA samples, each containing 50*µ*l of DNA at a concentration of 100 ng *µ*l/1, representing various chickpea genomes, were sent to Triticarte Pty. Ltd., a commercial service provider located in Australia (http://www.triticarte.com.au). In the genotyping process, 185 out of the initial 204 genotypes were successfully genotyped. Following this, the genotypic data of these 185 chickpea accessions were employed for the subsequent GWAS analysis. These genotypes were subjected to genotyping using the Chickpea SNP Panel Version 1.0. to ensure the reliability of the data, SNPs with a minor allele frequency (MAF) lower than 0.05 and a call rate below 90% were systematically excluded from the dataset using Tassel software. After rigorous sample quality control procedures, we identified a set of 1344 SNPs characterized by a proportion of missing alleles of 0.05 and a heterozygous proportion of 0.0097. These markers have been deemed robust genetic indicators within the chickpea genomes (**Figure 1**).

### 2.4 Population structure, clustering analysis, GWAS analysis, LD analysis, and haplotype analysis

Chickpea genotype diversity indices were examined using samples from 13 different countries in Europe, Asia, and Africa. The poppr package (v2.8.6) in the R environment was used to perform the analysis (Kamvar et al., 2014). It was used to discover indices such as the standard errors (SE), Shannon-Wiener index (H), heterozygozygosity (HExp), index of association (IA), and standardized index of association (rbarD).

Population structure analysis provides insight into genetic variation in chickpeas that has evolved through evolutionary processes such as genetic drift, demographic history, and natural selection (Andam et al., 2017). The LEA package was used to determine the genetic structure of 185 chickpea genotypes. This package utilizes two classical approaches, namely principal component analysis (PCA) and admixture analysis using sparse nonnegative matrix factorization (SNMF), to predict population genetic structure (Frichot and François, 2015). These methods calculate least-squares estimates of ancestry proportions and ancestral allelic frequencies for K populations ranging from 1 to 10. The Q-matrix (LEA output) representing the recorded ancestry proportions was investigated further to reveal the subpopulation percent using R calculations and further displayed in a bar-plot (Frichot et al., 2014).

SNPRelate is a high-performance computing R tool used for accelerating principal component analysis (PCA), and kinship analysis (Zheng, 2013). PCA analysis was conducted utilizing the snpgdsPCA, a function dedicated to assessing the genetic relatedness among accessions and generating the principal components (PCs) from the genotypic data. Subsequently, the snpgdsIBS function was employed to unveil the Identity by State (IBS) coefficients, elucidating the genetic similarity between individuals based on their genomic data. These IBS coefficients were then organized into a kinship matrix, shedding light on the genetic relationships within the studied population. This methodological approach allowed for a comprehensive exploration of the genetic structure and relatedness among individuals in the investigated cohort.

To identify markers associated with traits in chickpea, we utilized the vcf2gwas tool (Vogt et al., 2021). We used this pipeline to perform GWAS analysis using GEMMA software using the linear mixed model (LMM) (Danecek et al., 2021; Vogt et al., 2022). To identify meaningful associations, we established two-tier thresholds. Initially, we set a criterion with a False Discovery Rate (FDR) below 0.1. Additionally, we applied a p-value threshold of -log10-pvalue ≥ 2.5 (corresponding to a p-value ≤ 0.003). It’s important to note that we considered associations significant only if they consistently appeared in a minimum of two distinct environments. This dual-threshold strategy ensures a rigorous and dependable identification of significant associations in our study. We utilized boxplot analysis to illustrate the influence of each allele value on the expression of the studied phenotypes. We further tailored Manhattan plots alongside Q-Q plots, illustrating the disparity between expected and observed p-values (-log10(p)) by fitting a line that follows a normal distribution to the markers. We employed LDheatmap for visualizing the pairwise linkage disequilibrium between some markers, as described by Shin et al (Shin et al., 2006). The SNP haplotype analysis was performed out using geneHapR, a statistical R package for gene haplotype statistics, phenotype association, and visualization (Zhang et al., 2023). It was performed using 18 significant variants on chromosome one. Additionally, we utilized violin-plot analysis to illustrate the allele effects for some significant markers on the studied traits.

The BLASTn search on the NCBI database (chickpea genome of GCA 000331145.1) (https://blast.ncbi.nlm.nih.gov) was performed to investigate the genomic locations of the significant SNPs in order to find thier corresponding genes. The STRING database (https://string-db.org/) was used to give additional insight into the scope of these genes’ interactions and potential functional roles. The SRplot software (https://www.bioinformatics.com.cn/en) was used to efficiently depict the results of these extensive analyses.

## 3 Results

### 3.1 Analysis of variance

The analysis of variance (ANOVA) revealed a highly significant differentiation (P< 0.001) among the three sources of variation: genotype (G), environment (E), and the genotype-environment (G x E) interaction, across all agronomic traits (**Table 1**). Notably, significant differences (P< 0.01) in genotypes were observed for height index (HI) and drought tolerance (DR) traits. Meanwhile, HI displayed no significant difference in the genotype-environment interaction. The environment emerged as the primary source of variability, accounting for the majority of variability in traits such as days to flowering (DFL), days to maturity (DM), plant height (PLH), number of branches (NB), nodules’ fresh weight (NFW), nodules’ dry weight (NDW), grain yield (GY), and biomass yield (BY) (**Table 1**). Following closely behind were genotype environment interaction (genotype x environment) and genotypes, both of which played an important role in shaping the observed variations. Remarkably, genotype accounted for an important part of variation, particularly with 63.8% for 100SW. In contrast, genotype-environment interaction was critical in height index (HI) and drought tolerance (DR), accounting for 36.6 and 38.8% of the variability, respectively.

**Table 1:** Variance components analysis (ANOVA) of the studied agronomic traits for the 204 chickpea genotypes in the field experiment conducted at two locations during the 2016 and 2017 post-rainy season.

Traits	Source of variation	d.f.	s.s	m.s.	%ss	F-pr
DFL	E	7	80430.87	11490.13	64.2	*<* 0.001
	G	203	17944.75	88.39	14.3	*<* 0.001
	G x E	1417	16438.39	11.6	13.1	*<* 0.001
	Error	1611	10526.16	6.53		
DM	E	7	458003.54	65429.07	94.5	*<* 0.001
	G	203	8421.47	41.48	1.7	*<* 0.001
	G x E	1417	10686.19	7.54	2.2	*<* 0.001
	Error	16007	8861.65	5.514		
PLH	E	7	140927.47	20132.5	54.4	*<* 0.001
	G	203	34552.71	170.21	13.3	*<* 0.001
	G x E	1417	43348.52	30.59	16.7	*<* 0.001
	Error	1607	37871.75	23.57		
NB	E	7	5173.94	739.13	35.7	*<* 0.001
	G	203	2803.48	13.81	19.3	*<* 0.001
	G x E	1383	3599.15	2.6	24.8	*<* 0.001
	Error	1498	3176.66	2.21		
NFW	E	7	5716.94	816.7	38.6	*<* 0.001
	G	203	2976.6	14.66	20.1	*<* 0.001
	G x E	1383	3528.71	2.55	23.8	*<* 0.001
	Error	1498	2891.45	1.93		
NDW	E	7	179.02	25.57	30.6	*<* 0.001
	G	203	91.22	0.22	15.6	*<* 0.001
	G x E	1383	171.27	0.45	29.2	*<* 0.001
	Error	1498	157.48	0.11		
GY	E	7	52894.01	7556.29	34.8	*<* 0.001
	G	203	25194.63	124.11	16.6	*<* 0.001
	G x E	1417	37312.31	26.33	24.5	*<* 0.001
	Error	1605	35738.92	22.27		
BY	E	7	451304.06	64472.01	52.4	*<* 0.001
	G	203	82337.76	405.6	9.6	*<* 0.001
	G x E	1417	169029.65	119.29	19.6	*<* 0.001
	Error	1605	160425.95	99.95		
100SW	E	7	7974.15	1139.16	3.5	*<* 0.001
	G	203	144524.15	711.94	63.8	*<* 0.001
	G x E	1417	38567.84	27.22	17	*<* 0.001
	Error	1605	36068.99	22.47		
HI	E	7	9.13	1.31	12.3	*<* 0.01
	G	203	7.82	0.038	10.6	*<* 0.001
	G x E	1417	27.13	0.019	36.6	ns
	Error	1605	28.51	0.017		
DR	E	7	370.45	52.92	6.9	*<* 0.004
	G	203	1246.37	6.14	23.2	*<* 0.001
	G x E	1417	2081.32	1.46	38.8	*<* 0.001
	Error	1607	1642.98	1.022		

### 3.2 Diversity analysis

Assessing genetic diversity within chickpea populations is crucial for identifying genetically distant accessions, which can be instrumental in breeding programs aimed at developing hybrids with desired traits. The Shannon-Wiener index, ranging from 1.09 to 3.55, signifies richness and abundance among chickpea groups. However, the heterozygosity index, varying from 0.005 in Turkey to 0.08 in Azerbaijan, indicates restricted genetic diversity within subpopulations. Several factors, such as geographic location or sample size, could contribute to this observed limitation. The rbarD values varied from 0.01 in Nepal and Azerbaijan to 0.2 in Turkey. Additionally, the overall rbarD for the entire chickpea population was 0.05, suggesting a limited potential for sexual reproduction within the population, as evidenced by the heterozygosity index (**Table 2**).

**Table 2:** Chickpea genotypic diversity indices.

Pop	SE	H	Hexp	Ia	rbarD
IND	8.88E-07	3.55	0.05	36.02	0.05
PAK	0	4.78	0.04	46.16	0.06
ITA	0	1.09	0.02	13.84	0.03
NPL	0	1.09	0.008	7.26	0.01
RUS	0	1.09	0.02	34.46	0.08
TUR	0	1.38	0.005	79.95	0.2
AZE	0	1.38	0.08	9.99	0.01
AUS	0	1.38	0.06	25.41	0.05
Total	1.39E-05	5.21	0.04	39.77	0.05

The analysis covers standard errors (SE), the Shannon Index (H), heterozygozity (Hexp), the index of association (Ia), and the standardized index of association (rbarD).

**Figure 2:**
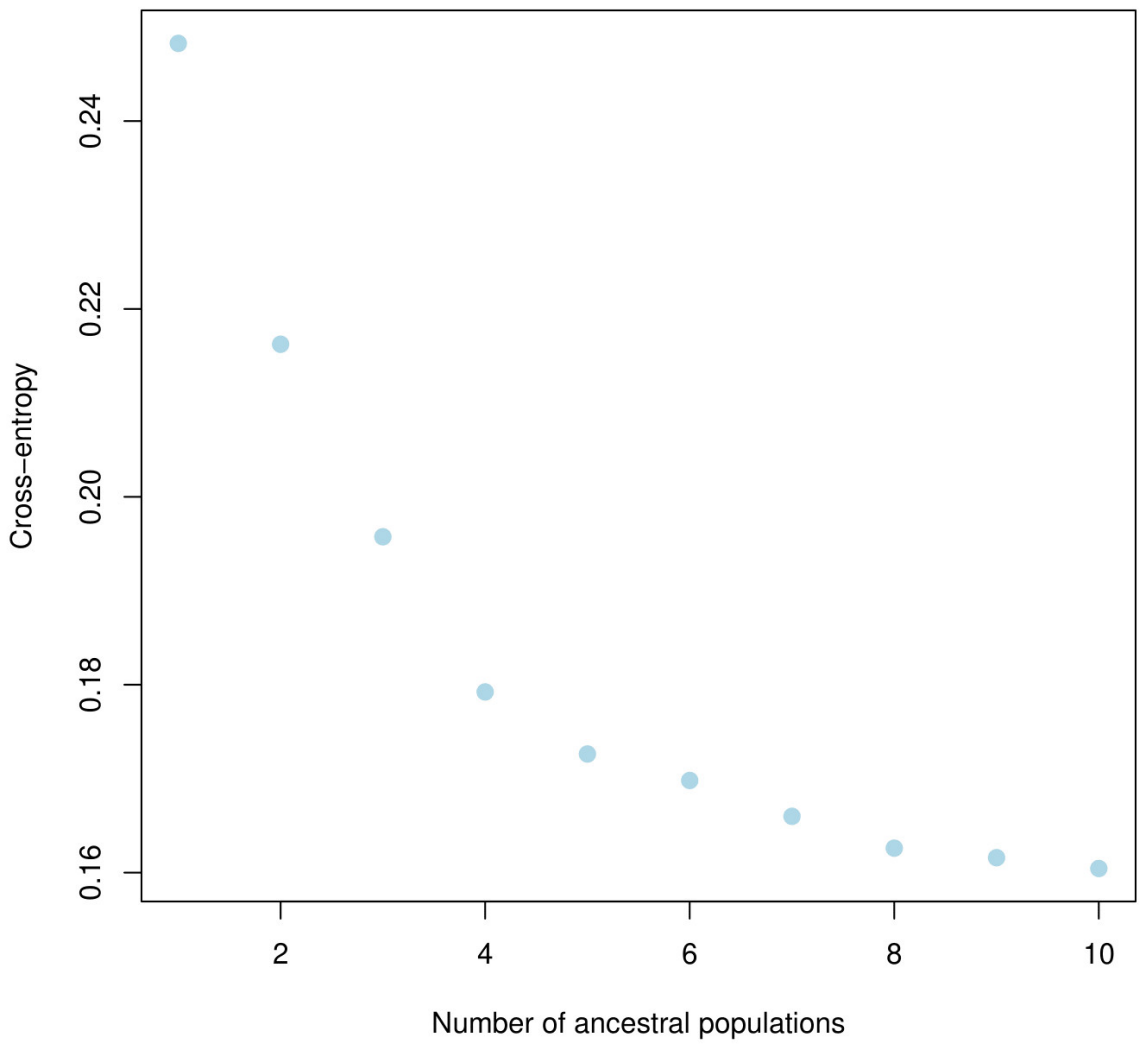
Five clusters were identified based on the curve of crossentropy versus the number of ancestral populations.

**Figure 3:**
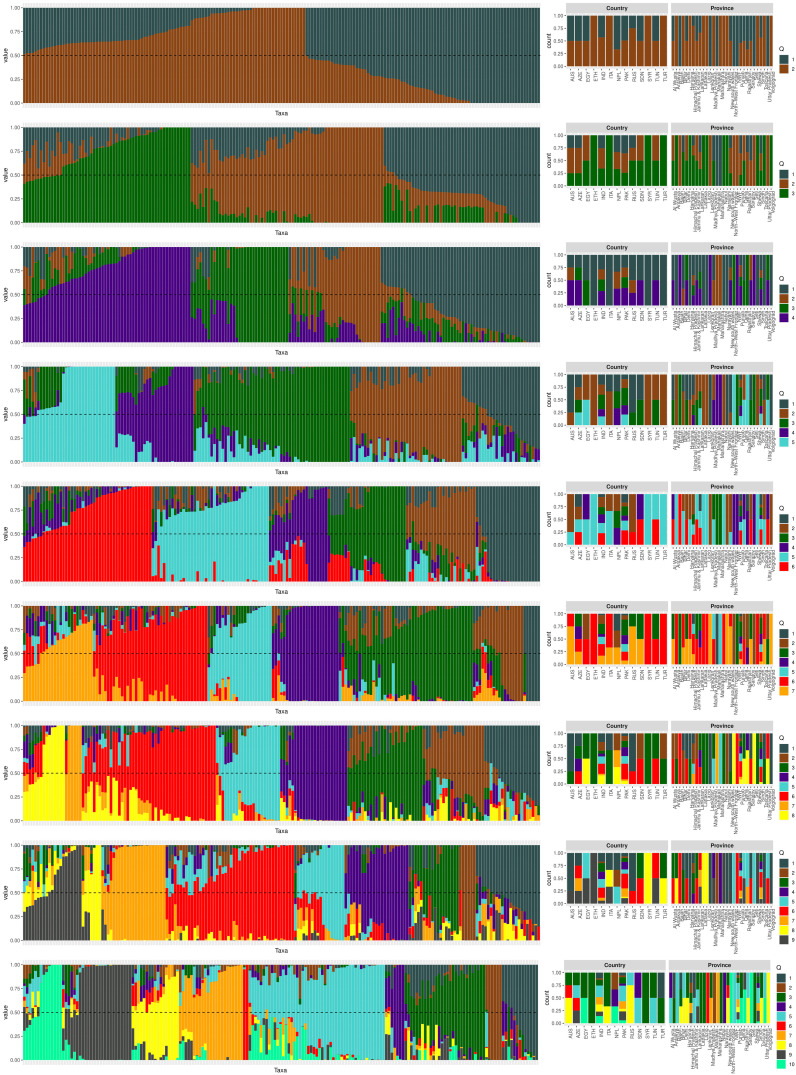
The population structure plot of the 185 genotypes shows that the ideal number of ancestries is 5 (K = 5), and K = 4 is also significant. Each vertical bar indicates a different genotype, while each color represents a country of origin.

**Figure 4:**
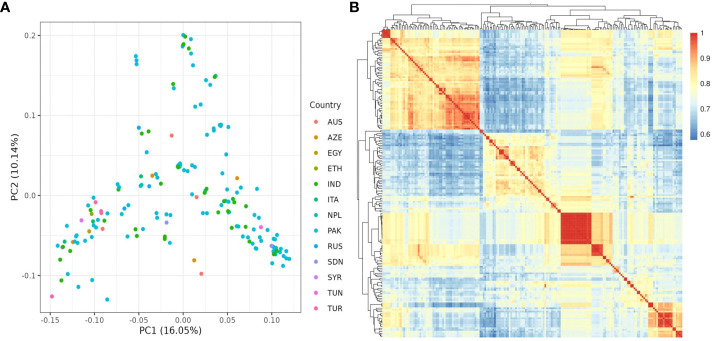
**(A)** PCA analysis of 185 chickpea accessions discovered 26.19 variances on the X (PC1) and Y (PC2) axes. **(B)** kinship matrix map demonstrating genetic diversity among chickpea accessions. It classified the accessions into four distinct groups.

The population structure of the 185 accessions was analyzed, and it was found that the optimal number of subpopulations, or K value, was 5, with 4 also being significant (**Figure 2**). This suggests that these accessions could be classified into five distinct genetic groups. Population Q1 contains 28 admixed accessions, representing 15% of the total, with the majority of these accessions being of Pakistani origin (11 accessions). Population Q2 comprises 40 accessions, which is 21.6% of all genotypes, with six pure and 34 admixed genotypes. The largest population, Q3, contains 56 accessions, with 21 pure and 35 admixed genotypes. Q4 has 28 accessions, of which 19 are admixed and nine are pure. Q5 is made up of 33 accessions (17.83%), with 18 pure and 15 admixed genotypes, and again, Pakistani accessions are the most common, with 25 genotypes (**Figure 3**). Cluster analysis was performed to confirm the structure analysis. PCA analysis revealed that the population could be divided into five groups with significant genetic variations among varieties (**Figure 4**). In addition, principal component analysis (PCA) revealed that the first two axes explained 26.19% of the variation.

### 3.3 SNP markers associated with chickpea traits in rainfed conditions

**Figure 5:**
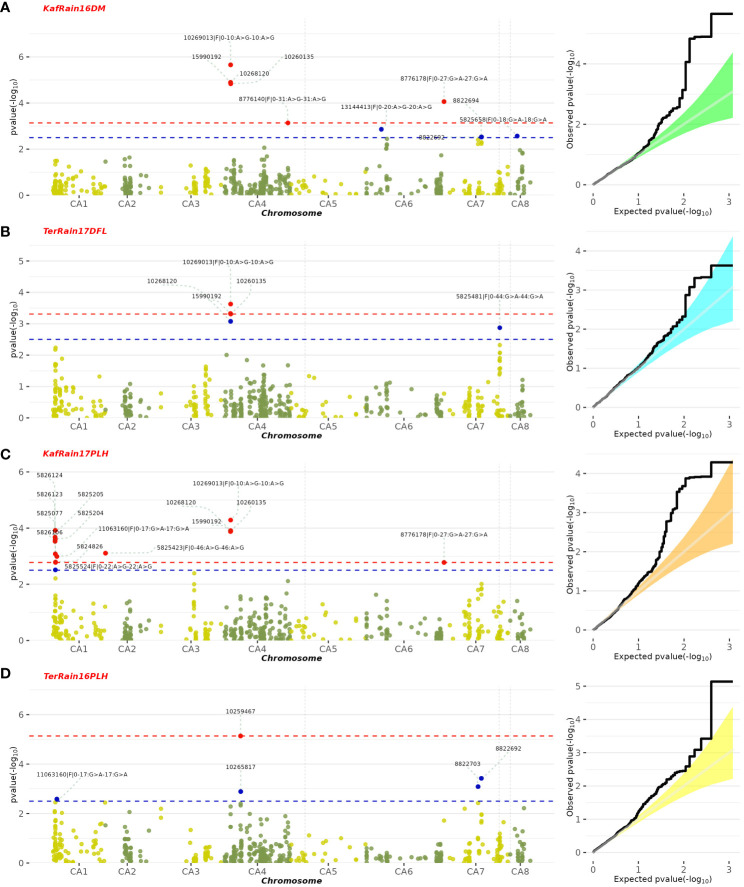
Manhattan plot illustrating SNPs linked to specific chickpea traits under rainfed conditions, along with their corresponding statistical significance represented by FDR. The featured GWAS results correspond to the following traits and years: **(A)** Rainfed-Kfardan DM in 2016, **(B)** Rainfed-Terbol DFL in 2017, **(C)** Rainfed-Kfardan PLH in 2017, **(D)** Rainfed-Terbol PLH in 2016.

**Figure 6:**
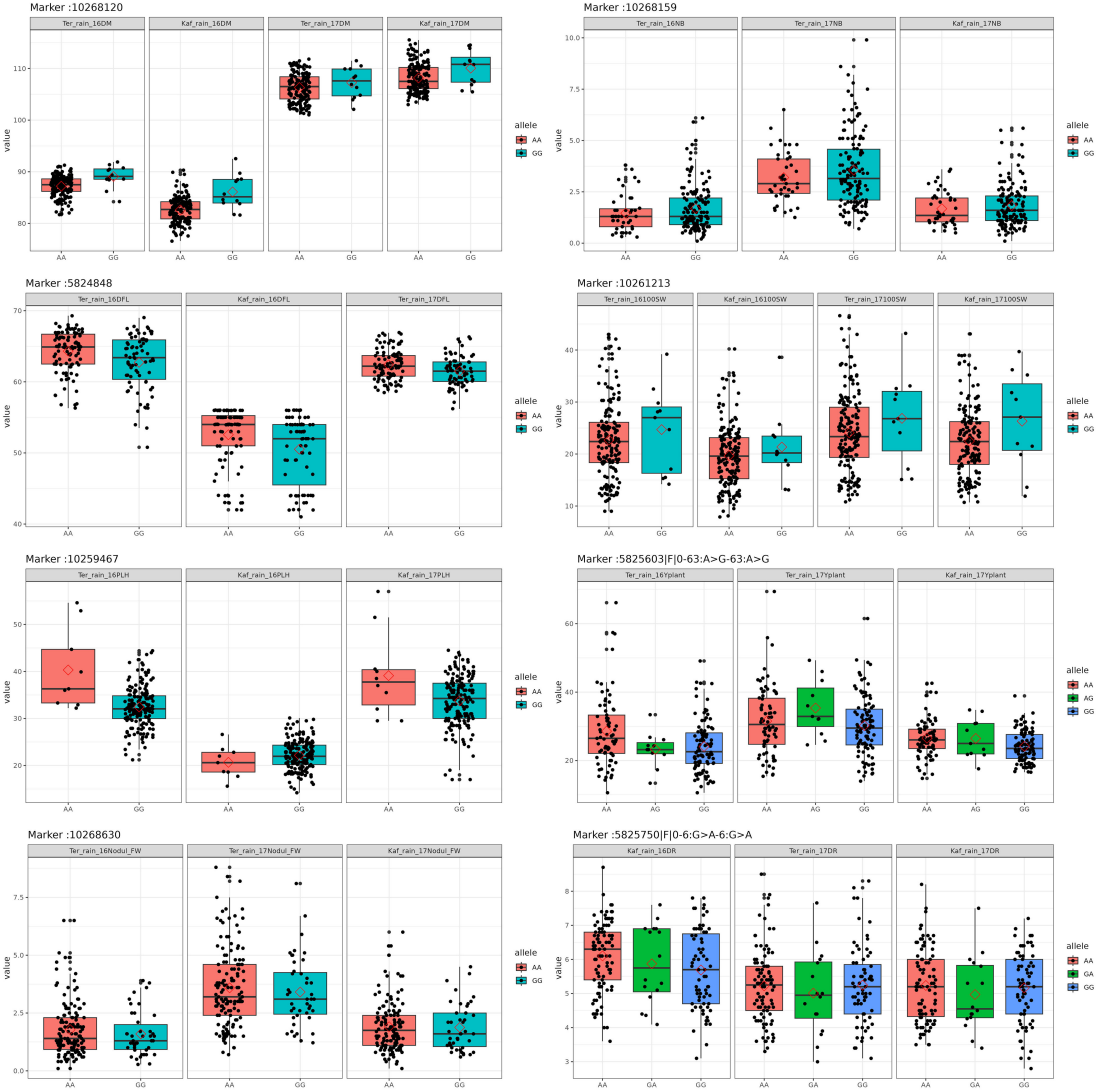
Box plots of the allele effects for the chickpea markers in rainfed conditions.

**Figure 7:**
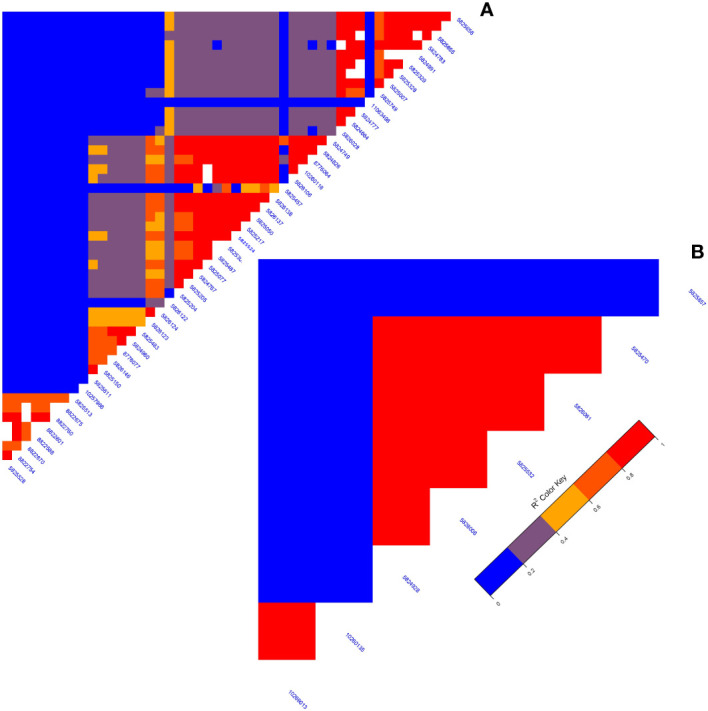
**(A)** The LD map of an area on chromosome one running from 3093579 to 1057324 reveals a high correlation of markers associated with many characteristics, particularly plant height. **(B)** The LD plot highlights associations between specific markers influencing diverse characteristics.

**Figure 8:**
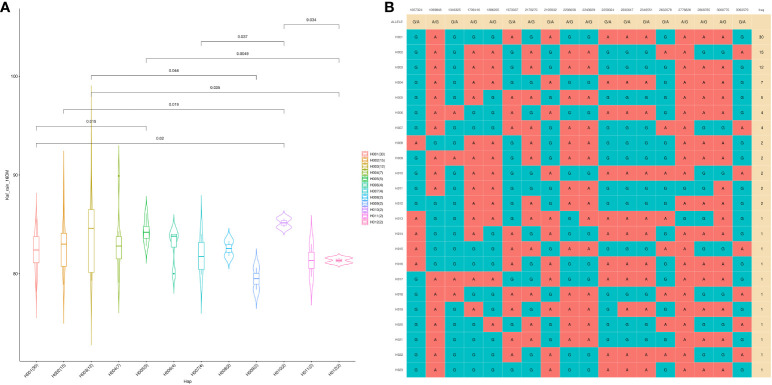
The haplotype analysis showing haplotype blocks comprise 18 significant SNP markers located on Ca1 **(A)**, with 23 distinct haplotype variants (H001–H023) observed across the analyzed populations, each occurring at different frequencies. **(B)** The violin plot illustrates the phenotype values associated with the 23 haplotype groups specifically in the Kfardan rainfed location.

A GWAS analysis was carried out to identify SNPs associated with 11 investigated variables in chickpeas, including morphological, yield, and drought tolerance (**Figure 5**, and **Supplementary Figures 3**). These SNPs were classified based on their relationships with chickpea characteristics in rainfed settings, rainfed and irrigated environments within the same trait, and those found in two or more traits. Initially, 11 SNPs showed relationships with chickpea-examined variables in rainfed conditions, with a high effect on chickpea traits (**Figure 6**). Additionally, the analysis of linkage disequilibrium (LD) highlighted two genomic regions with notably strong LD, suggesting significant interconnections among several investigated traits (**Figure 7**). Haplotype analysis of 18 significant related SNPs on chromosome one, revealed 23 haplotype blocks, their frequencies, positions, and alleles. Hap1,2,3,4,5 (H001-H005) had a significant association with the studied trait (**Figure 8**).

SNP 10268120, situated on chromosome 4 at location 8115377, for example, showed a strong correlation with chickpea maturity, with a P-value of 1.2x10^−5^ and an FDR of 2x10^−3^. Furthermore, SNP 5824848, which is located on chromosome 1 at location 7866973, has been discovered as a determinant of chickpea blooming as it exhibited the greatest significant p-value (6x10^−4^) and associated statistical significance (FDR value of 3x10^−1^). Remarkably, SNP 10259467, situated on Chromosome 4 at location 16564317, demonstrated strong relationships with plant height, with very low P-values (7.2x10^−6^) and statistical significance, as evidenced by an FDR value of 5x10^−3^. SNPs 10268630, 15990164, and 10268159 on chromosomes 4, 7, and 7 at 27746660, 56720159, and 28648217 were also linked to chickpea nodule properties including fresh weight, dry weight, and biomass. These relationships had p-values of 3x10^−4^, 1x10^−3^, and 6x10^−3^, with FDRs of 4x10^−2^, 5x10^−1^, and 6x10^−1^, respectively (**Table 3**). SNP 10261213 on Chromosome 4 at location 5406817 had a significant P-value of 7x10^−3^ and an FDR of 8x10^−1^ for the 100SW trait. SNP 5825603 (Chromosome 3 and location 49807823) has a strong correlation with chickpea biological yield, with a p-value of 4x10^−3^ and an FDR of 6x10^−1^. SNP 5826170 (chromosome 7 and location 47381973) was connected to the height index trait in chickpea with a p-value of 3x10^−4^ and an FDR of 2x10^−1^. SNP 5825750 (chromosome 1 and location 7699696) had a significant p-value of 4x10^−4^ and an FDR (1x10^−1^), indicating that it is near a gene with a potential link to drought tolerance stress.

**Table 3:** The significant markers related to chickpea characteristics in rainfed conditions.

Marker	Trait	CHR	POS	P-VALUE	FDR	Gene	ID
SNP-10268120	DM	CA4	8115377	0.000012	0.002	*CPN60-2*	LOC101510250
SNP-5824848	DFL	CA1	7866973	0.0006	0.3	*hsp70*	LOC101514822
SNP-10259467	PLH	CA4	16564317	0.0000072	0.005	*GDSL(GELP)*	LOC101511651
SNP-10268630	NFW	CA4	27746660	0.0003	0.04	*AHL16*	LOC101509190
SNP-15990164	NDW	CA4	56720159	0.001	0.5	*NAT3*	LOC101493208
SNP-5826170	HI	CA7	47381973	0.0003	0.2	*FAB1B*	LOC101496798
SNP-5825750	DR	CA1	7699696	0.0004	0.1	*bZIP*	LOC101509540
SNP-5825172	GY	CA1	7475256	0.0005	0.2	*GL21*	LOC101503879

Furthermore, the greatest determinant of chickpea grain yield was SNP 5825172, which was discovered on chromosome 1 at position 7475256, with a p-value of 5x10^−4^ and an FDR value of 2x10^−1^. These findings contribute to a deeper understanding of the genetic factors influencing chickpea traits in rainfed environments, highlighting potential targets for further breeding and improvement programs (**Table 3**).

### 3.4 SNP markers associated with drought-related traits in two or more environments within the same trait

The GWAS findings were further investigated to reveal 11 SNPs that are common in at least two environments within the same trait and therefore could be employed as a marker for this trait. SNP 15990192 (p-value of 1.2x10^−5^ and 2x10^−3^ FDR) has been investigated in the irrigated and rainfed environments of the Kfardan region as a marker for chickpea maturity. A marker for chickpea flowering could be SNP 10260135 (4x10^−4^ p-value and 1x10^−1^ FDR), which was investigated in four rainfed and irrigated environments in Kfardan and Terbol. SNP 11063160 was discovered in three habitats in the Kfardan and Terbol regions in 2016 and 2017, with a p-value of 1x10^−3^ and an FDR of 6x10^−2^. This suggests that it could be useful as a plant height indicator. At the same time, SNPs 5825455 (p-value: 3x10^−4^, FDR: 4x10^−2^), 5825655 (p-value: 2x10^−3^, FDR: 6x10^−1^), and 5825419 (p-value: 4x10^−3^, FDR: 5x10^−1^) emerged as promising markers for different chickpea nodule characteristics (fresh weight, dry weight, and biomass) (**Supplementary Table 4**). Notably, SNP 5826197, related to the 100SW feature, confirmed its marker potential by being detected in five habitats in Kfardan and Terbol, both irrigated and rainfed. SNP 5824850 is being studied as a marker for chickpea biological yield in the rainfed and irrigated environments of Kfardan and Terbol in 2016 and 2017. SNP 11064344 in the height index trait was explored in 2017 within the Kfardan rainfed environment and in 2016 in the Terbol irrigated environment. SNP 5825205 was discovered in irrigated environments in Kfardan and Terbol during 2017 and could be a marker for drought tolerance. Since it was studied in three different environments, SNP 23870854 (1x10^−3^ p-value and 4x10^−1^ FDR) could be referred to as the grain yield marker.

### 3.5 SNP markers associated with drought-related traits in two or more different traits

**Figure 9:**
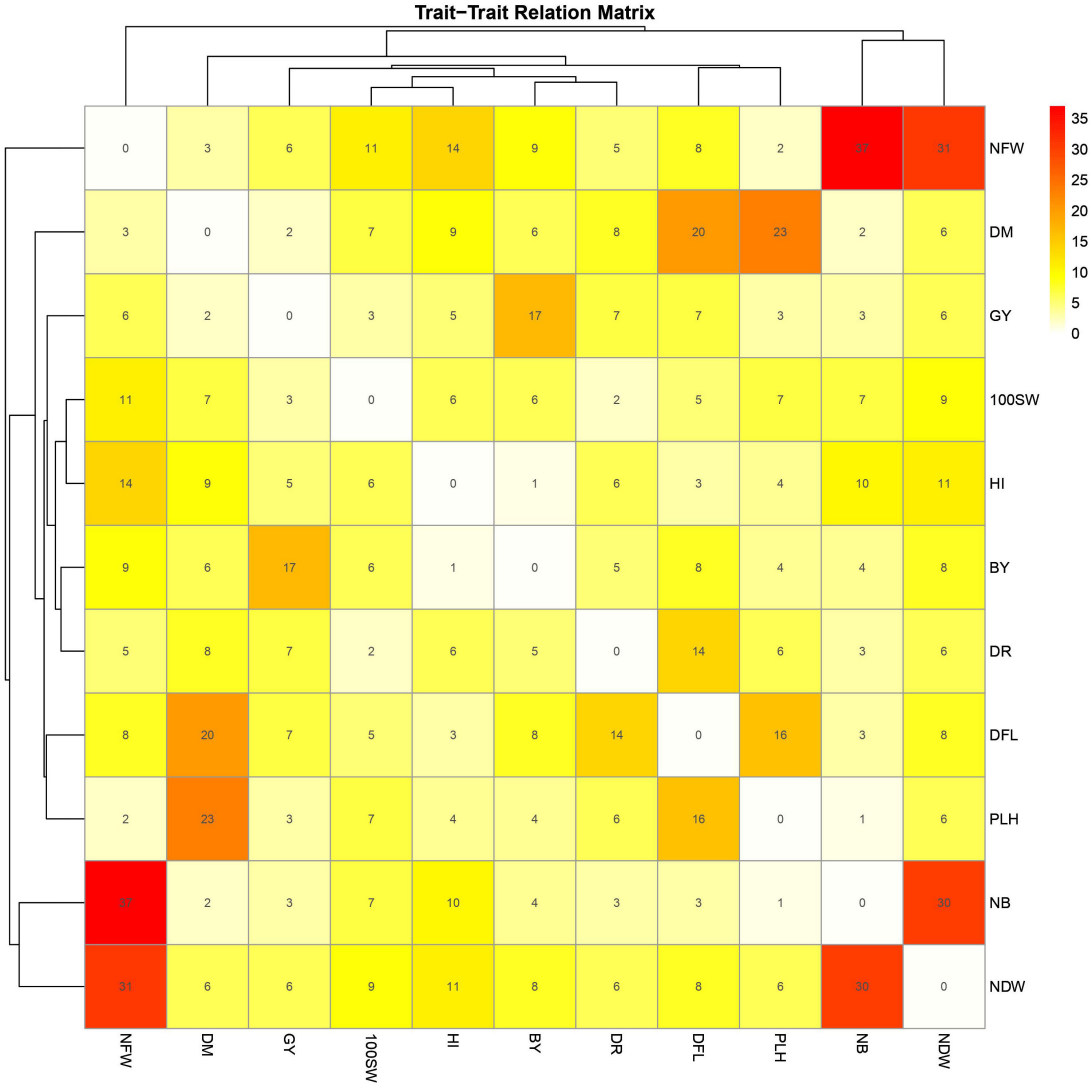
Heatmap matrix depicting shared markers among 10 traits (100SW, BY, GY, NDW, NFW, NB, PLH, DFL, DM, and DTS) with a significance threshold of p-value< 0.001. Only markers that are detected in at least two environments for the same trait are included. The marker numbers are shown in the center of each square, representing shared markers between two traits.

The GWAS results were fully examined in order to identify markers that were common across many traits. Six marker SNPs with notable p-values and statistical significance (FDR) were discovered in this study. On chromosome 4, location 8136235, SNP 10269013 (p-value: 2.2x10^−6^, FDR: 1x10^−3^) emerged as a significant marker related to five variables, including day of blooming, maturity, drought tolerance, nodule fresh weight, and plant height (**Figure 5**; **Supplementary Figure 3**). SNPs 10258537 and 5824976 (located on chromosomes 4 and 6 at locations 28648151 and 20524867) were found to be linked with nodule characteristics (fresh weight, dry weight, and nodule biomass) with p-values of 1x10^−4^ and 3x10^−3^, and FDRs of 4x10^−2^ and 4x10^−1^, respectively. SNP 11063680 (p-value: 2x10^−4^, FDR: 8x10^−2^) on chromosome 2 at position 21148420 was associated with 100SW, flowering, and maturity days (**Figure 9**; **Supplementary Table 5**). Furthermore, SNP 5824555 (located on chromosome 2 at location 7717206) demonstrated significance for days of flowering and grain yield attributes, with a 1x10^−4^ p-value and a 7x10^−2^ corresponding FDR. Finally, SNP 8776178 was found to be associated with chickpea maturity, height index, and plant height (p-value: 8.6x10^−5^, FDR: 1x10^−2^).

### 3.6 Gene enrichment analysis

**Figure 10:**
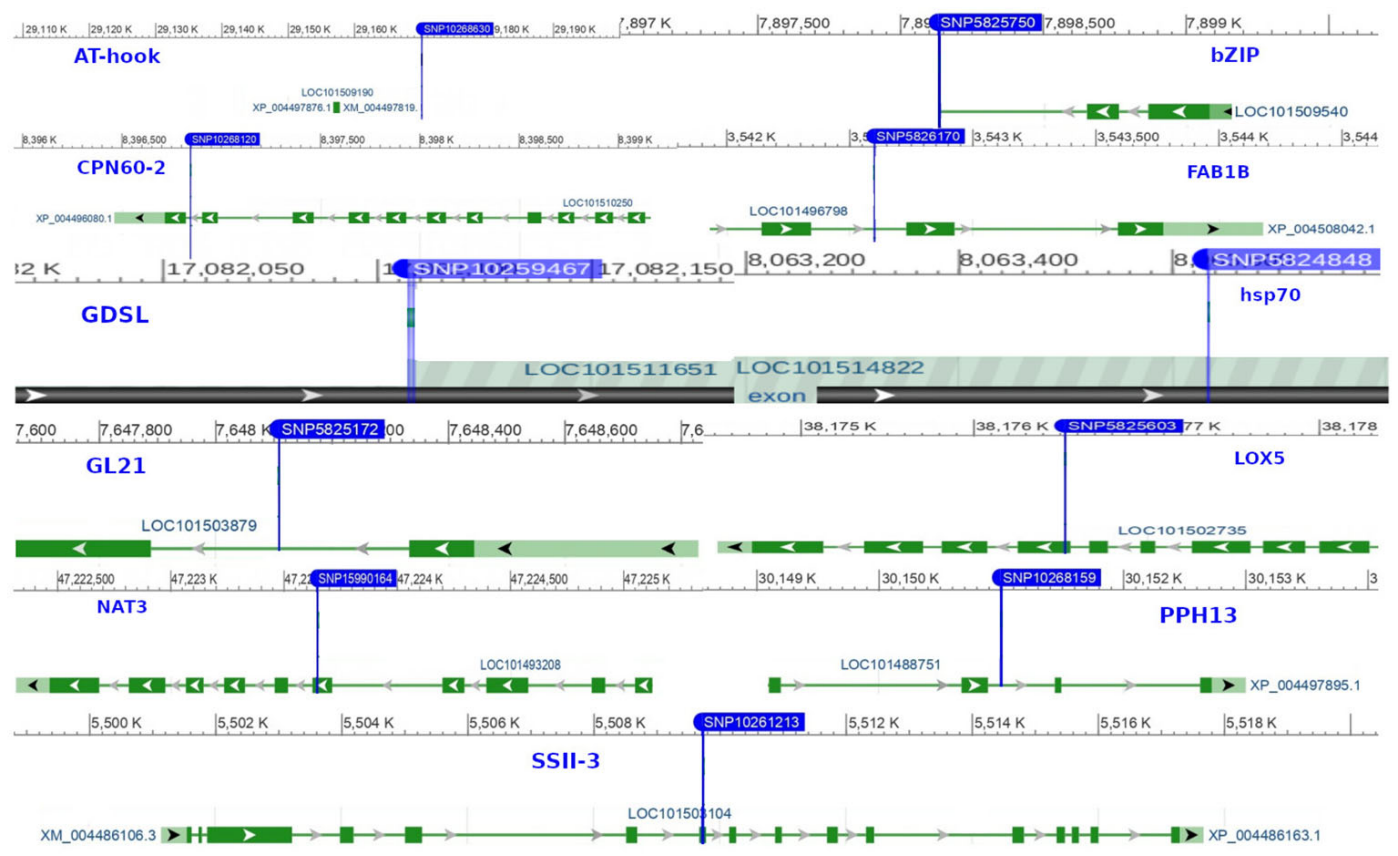
The 11 key markers and genes associated with chickpea in rainfed environments.

SNP localization was performed to uncover the genetic basis of the observed phenotypes. Through the annotation of significant markers, we identified 28 candidate genes involved in regulating biological processes, cellular components, and molecular functions in chickpea (**Supplementary Table 4**). Genes such as *CPN60-2*, *hsp70*, *BSD*, and *NUP1* harbored primary marker SNPs related to chickpea maturity and flowering, playing crucial roles in auxin synthesis, vegetative growth, leaf senescence, and flowering organ development (**Figure 10**). Several SNPs associated with studied agronomic traits were found within genes strongly linked to plant height and nodule characteristics, such as *GDSL(GELP)*, *AHL16*, *NAT3*, and *PAP13*. Additionally, genes like *SSII-3*, *LOX5*, *GL21*, *SCP*, and *ABP19a* are related to chickpea 100SW, biological yield, and grain yield.

**Figure 11:**
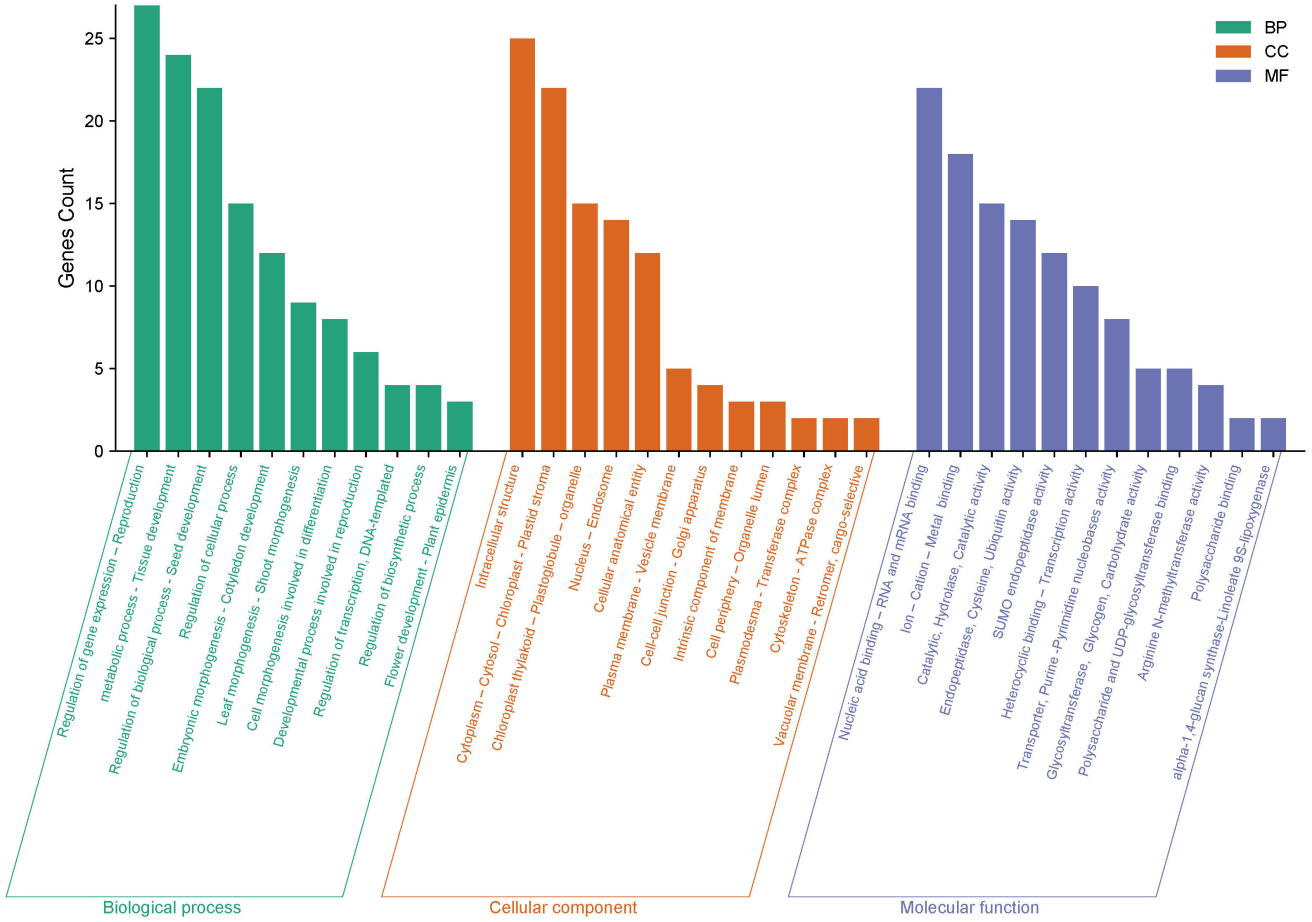
The gene ontology (GO) analysis of 28 genes impacting chickpea characteristics was carried out in three categories: biological process, cellular component, and molecular function. The Y-axis represents the number of genes, while the X-axis depicts the function of the genes.

The gene ontology analysis revealed that these genes are involved in a variety of biological processes, including metabolic processes, seed development, leaf morphogenesis, and tissue development. These genes contribute to the cytoplasm, cytosol, chloroplast-plastid stroma, cytoskeleton, and cellular entity. Furthermore, it was discovered that these genes encode essential DNA and RNA binding, endopeptidase, purine and pyrimidine nucleobase activity, polysaccharide binding, and phospotransferase activity, among other molecular functions. These findings provide valuable insights into the potential roles of these genes in regulating chickpea traits and may inform future research efforts aimed at improving crop productivity and resilience (**Figure 11**).

### 3.7 Discussion

Chickpea is the second-most important crop, with a high economic and nutritional value. It is a low-cost source of dietary proteins, carbohydrates, vitamins, micronutrients, and fats required for human nutrition (Gaur et al., 2016). In this investigation, the assessment of chickpea genotypes across diverse environments aimed to identify those with stable and broad-based resistance to drought stress in different locations across Lebanon. Multi-environment testing revealed distinctive responses to drought among the evaluated genotypes. The environment and its interactions had a significant impact on how these genotypes responded to drought stress. The significant interaction effects between chickpea genotype and environment underscored the variability of genotypes across diverse geographical locations. However, it is essential to consider that different genotypes might also exhibit varied responses to distinct environmental conditions. For the majority of the studied traits, the analysis revealed that environmental effects were more pronounced than the genotype-environment (G x E) interaction (**Table 1**). This observation suggests that there is significant variability among genotypes and environments, implying the possibility of selecting stable genotypes (Chobe and Ararsa, 2018; Erdemci, 2018). Furthermore, the greater impact of the G-E interaction compared to genotypes (**Table 1**) suggests that genotypes respond differently to changes in environmental conditions. Overall, these findings contribute valuable insights into the interaction of genotypes and environmental factors, providing a foundation for the selection of drought-resistant and stable chickpea genotypes.

The adoption of novel breeding approaches holds promise for overcoming current barriers in breeding programs. Traditional methods fall short of meeting global needs projected to reach 10 billion people by 2050, posing a significant challenge for breeders (Hickey et al., 2019). Next-generation sequencing (NGS) technologies have emerged as a promising pathway for breeders, providing invaluable genomic resources to discern alleles and haplotypes linked with agronomic traits in chickpeas (Roorkiwal et al., 2020; Ibrahim and Ibrahim, 2022). DART (Diversity Arrays Technology), a method based on methyl filtration and next-generation sequencing platforms, is one of the notable SNP genotyping techniques (Sansaloni et al., 2011; Kilian et al., 2012). It is also a cost-effective solution for marker discovery and assay development (Kilian et al., 2003). Using this technique and further filteration, 1344 genetic markers for the 185 global chickpeas were identified. The SNP density analysis revealed that SNPs are distributed across all chromosomes, with chromosome 4 being the most densely packed and chromosome 8 being the least (**Figure 1**).

The genetic diversity of chickpea germplasm could provide important information for selecting effective parental breeding strategies as well as a better understanding of natural variations in phenotypic traits and their genetic background (Raina et al., 2019). To address this issue, three diversity analyses were carried out: population structure analysis, PCA, and the kinship matrix. We conducted population structure analysis based on the geographical locations of the chickpea accessions (**Figure 3**). The analysis identified five ancestries with specific admixtures, indicating potential variations in the origin of our chickpea population. This aligns with a previous study on Desi and Kabuli chickpea genotypes, which emphasizes the importance of geographic origin and adaptive environments in genotype clustering (Basu et al., 2018).

Further investigation revealed that most sub-populations were admixed, with the exception of specific accessions in groups 5 and 3, which could be useful in breeding programs to produce hybrids with desirable traits. It’s noteworthy that admixing among populations can be attributed to various factors, including different origins of germplasm derived from various countries worldwide as well as their involvement in breeding programs leading to changes in genetic makeup. These findings are supported by the pure and minimally admixed accessions studied by Bajaj et al. (2015) in wild Cicer species (*C. reticulatum* and *C. echinospermum*). Principal Component Analysis (PCA) was employed to uncover relationships between structural groups (**Figure 4A**). Geographic data indicated only minor differences among chickpea genotypes, supporting the structure analysis results that classified the germplasm into five admixed groups. The first two axes (PC1-PC2) explained 26.19% of the total variation. While Pakistani and Russian accessions stood out genetically from the majority, they did not form a distinct group when grouped together. The cluster analysis, utilizing the kinship matrix, further divided the chickpea genotypes into four groups. These groups exhibited varying degrees of kinship, with low kinship degrees represented in cyan and high degrees in red (**Figure 4B**).

In recent years, the GBS (Genotyping by Sequencing) approach, including GWAS, has become crucial for pinpointing SNPs in chickpeas, helping us understand genetic diversity and identify genomic variations linked to diseases or specific traits (Roorkiwal et al., 2020). This study utilized GWAS on 11 chickpea features to reveal significant details about SNPs that could serve as markers for these traits. Notably, the day of maturity was the trait most affected by SNPs (**Supplementary Figure 4**), with 98 marker SNPs, and it showed a strong correlation with the flowering trait, sharing 20 marker SNPs (**Supplementary Tables 5**, **6**). This suggests a potential integration of genes governing both traits. The LD analysis supports this, showing a robust link between SNP 10260135 associated with days to flowering (DFL) and SNP 10268120 linked to chickpea maturity under rainfed conditions (**Figure 7B**). These findings align with previous studies, suggesting that alleles for double podding and early flowering can enhance early maturity in chickpeas, minimizing the risks of excessive canopy development in specific locations (Anbessa et al., 2007). SNP 10268120 is one of the significant markers associated with flowering under rainfed conditions (**Figure 10**). It was discovered on the *mitochondrial-like chaperonin CPN60-2*, a protein that influences the folding of chloroplast protein polypeptides (Wu et al., 2020). Additionally, research on cowpeas has shown that it is a target for the development of new genotypes and auxiliary molecular markers for water stress-resistant breeding programs (Lima et al., 2019).

Flowering days represent a crucial trait in chickpeas and the broader plant kingdom. Our study identifies 73 SNP markers in chickpeas associated with flowering (**Supplementary Table 3**). Existing research has illuminated the intricate relationship between plant flowering time, seed size, and drought tolerance (**Supplementary Table 6**). Plants, including chickpeas, exhibit an evolutionary adaptation to adjust their flowering time in response to stress, optimizing reproductive success and maturity, especially under conditions like drought (Kazan and Lyons, 2016). Our findings affirm this association, emphasizing the highest correlation between flowering and maturity, with 14 markers shared between flowering and the drought trait and 5 markers shared between days of flowering and 100SW (**Supplementary Table 5**). The LD analysis further validates these results, revealing a strong linkage between SNP 10260135, associated with DFL, and other markers related to 100SW and drought (SNP 10268120-SNP 10269013) (**Figure 7B**). Notably, SNP 10260135, a rainfed and irrigated marker for DFL, is also associated with the *nuclear pore complex protein (NUP1)*. This nucleoporin is one of eight that participate in a variety of plant life cycle processes such as nodulation, flowering, pathogen interaction, hormone signaling, and cold response (Tamura et al., 2010). In rainfed conditions, another significant DFL marker is SNP 5824848. It is related to the *heat shock cognate 70 kDa (hsp70)*, a gene in the heat shock protein family (HSP) that is important for heat avoidance and normal biological processes in plants (Aghaie and Tafreshi, 2020). It is also required for flower opening under normal or mild heat stress conditions (Chen et al., 2019).

Plant height in chickpeas is influenced by 93 SNPs; additionally, it is primarily influenced by markers after maturity (**Supplementary Tables 5**, **6**). This is consistent with earlier studies showing multiple loci control plant height in different crops. The multi-locus control of plant height is influenced by both genetic and environmental factors, although specific details are not fully understood (Yang et al., 2021). In soybeans, 19 loci associated with plant height under drought stress have been identified (Yang et al., 2021). The linkage analysis identifies a region on chromosome one, from position 3093579 to 1057324, that is rich in markers associated with plant height (SNP 5824826, SNP 5825204, SNP 5826123, and SNP 5826124) (**Figure 7A**). This region also shows strong linkage with markers (SNP 5825611, SNP 5825655, SNP 11063498) related to traits such as DR, NDW, and DM. Plant height shows a positive correlation with phenological traits (time to flowering, podding, and maturity), indicating that early flowering leads to shorter plant height (Sundaram et al., 2019). Our study further identifies a positive correlation between plant height and the days of flowering and maturity, involving 16 and 23 markers, respectively (**Figure 9**). SNP 10259467 is a significant marker related to the chickpea plant height trait in rainfed conditions. This marker is located near *GDSL esterases and lipases (GELPs)*, a group of lipolytic enzymes that hydrolyze various lipidic substrates (**Figure 10**). Increasing research has confirmed their roles in both vegetative and reproductive development, as well as plant metabolism (Shen et al., 2022).

Nodule characteristics are significant traits in chickpeas (**Supplementary Figures 8**-**10**). Inoculating with competitive rhizobia improves nodule formation, thus enhancing chickpea yield (Gul et al., 2014). We discovered that 66, 68, and 66 marker SNPs influence nodule characteristics, including NB, NDW, and NFW, with many markers shared among them (**Supplementary Tables 6**–**9**). Notably, these nodule traits share 7 and 6 markers with 100SW and grain yield, respectively (**Supplementary Table 5**). While studies emphasize the importance of nodule traits in relation to grain yield, there is some inconsistency regarding the positive correlation between nodule characteristics and 100SW. Some studies highlight a positive correlation (Istanbuli et al., 2022), while others report a nonsignificant negative relationship between nodule plant and seed yield (Gul et al., 2014). However, it has been suggested that 100SW is related to fertilizer applications and seed size; specifically, plants germinating from large seeds exhibit a greater number of pods and seeds, higher seed weight, and increased seed yield (Erdemci et al., 2017). Nodule characteristics are associated with SNPs 10268630 and 15990164, which are located on the *AT-hook motif nuclear localized protein 16 (AHL16)* and the *nucleobase-ascorbate transporter 3 (NAT3)*, respectively. AT-hook Protein, an ancient transcription factor found in all plants, plays a crucial role in various plant growth and development processes (Zhang et al., 2022). It positively regulates arabinogalactan protein expression for nexine formation in Arabidopsis (Jia et al., 2015). Our findings support previous research, suggesting its indirect association with nodule formation by regulating metabolic processes in root cells. The *nucleobase-ascorbate transporter 3 (NAT3)* gene belongs to the NAT (Nucleobase-Ascorbate Transporters) gene family and is also linked to plant growth, development, and stress resistance (GUO et al., 2022). It is one of sixteen identified transporters in soybean nodules that respond to phosphorus (P) deficiency (Xue et al., 2018).

Drought is a critical factor affecting plants, especially those in dry regions, and stands as a major constraint to chickpea production, particularly when accompanying high-temperature stress (Devasirvatham and Tan, 2018). Our research has discovered 49 SNP markers linked to drought (Devasirvatham and Tan, 2018) (**Supplementary Tables 6**, and **10**). SNP 5825750 is a critical marker associated with drought in rainfed areas (**Figure 10**). It is close to bZIP transcription factors, which play an important role in regulating essential plant processes like pathogen defense, signaling in response to light and stress, seed maturation, flower development, and responses to various environmental stresses (Jakoby et al., 2002; Alves et al., 2013). Another significant marker, SNP 5825205, is linked to drought resistance in chickpea under both rainfed and irrigated conditions. This marker is located on *polyphenol oxidase A1 (PPO-A1)*, a chloroplastic-like enzyme with important functions but the potential to harm the plant. PPO enzymes catalyze the oxidation of mono- and o-diphenols to o-diquinones in the presence of oxygen. This process is linked to oxidative browning seen in plant senescence, wounding, and pathogen responses (Thipyapong et al., 2004; Goel et al., 2015). PPO susceptibility increases with antisense suppression, and overexpression enhances resistance to Pseudomonas syringae, a bacterial pathogen. It has been reported that suppressing PPO improves plant water relations, delays photoinhibition, and prevents photooxidative damage during plant water stress in tomatoes (Thipyapong et al., 2004). Despite limited studies on *PPO-A1* in chickpeas, our research contributes to linking these markers with drought tolerance. We observed integration among drought-related markers and other traits, with 14 SNP markers shared between drought and flowering day traits. Furthermore, it shares 8 and 6 SNPs with the traits of day of maturity and plant height, respectively. The linkage between SNPs 5825205, 11063498, and 5825611, which are related to drought, maturity, and chickpea height, may also support this high integration (**Figure 7A**).

This study employed the evaluation of various phenotypic traits in chickpea genotypes, offering a thorough understanding of their characteristics and enabling a thorough analysis of genes affecting different traits. The experiment was conducted across two diverse regions and over two seasons, enhancing the robustness of the results by capturing the variability due to environmental factors and providing reliable data for analysis. However, it is worth noting that the study involved a relatively small number of chickpea accessions and breeding lines, which could be expanded to encompass a more representative coverage of the genetic diversity present in the chickpea population. Additionally, including more diverse geographic locations would provide a broader understanding of chickpea genotype performance across different agro-climatic conditions. Notably, we identified several genes potentially important for chickpea drought tolerance based on their associated SNPs with essential agronomic traits under drought conditions. However, further investigation is necessary to validate these findings functionally, such as through gene expression analysis or gene knockout studies, to provide additional evidence for the role of specific genes in controlling chickpea traits. Future studies should aim to improve upon these findings, and we encourage chickpea researchers to consider these potential results as a blueprint for breeding resilient genotypes with enhanced drought tolerance.

## Data availability statement

All data utilized in this article are accessible on the Zenodo database through the link : https://doi.org/10.5281/zenodo.10546540.

## Author contributions

TI: Resources, Validation, Data curation, Conceptualization, Methodology, Writing – review & editing. AEN: Investigation, Data curation, Conceptualization, Formal analysis, Visualization, Software, Methodology, Writing – original draft, Writing – review & editing. MAE-M: Investigation, Validation, Data curation, Conceptualization, Methodology, Writing – review & editing. ST: Investigation, Validation, Data curation, Visualization, Software, Methodology, Conceptualization, Writing – review & editing. AMA: Project administration, Validation, Supervision, Software, Formal analysis, Visualization, Writing – review & editing, Conceptualization, Data curation. AH: Project administration, Supervision, Conceptualization, Data curation, Resources, Investigation, Writing – review & editing, Funding acquisition.

## Supplementary material

Supplementary Table 1 Geographic Origin and Accession Numbers of the 204 Germplasm Samples Utilized.

Supplementary Table 2 The evaluated phenotypic traits for the 185 chickpea genotypes.

Supplementary Table 3 Results from the GWAS analysis for all traits were derived using SNP genotyping data.

Supplementary Table 4 Related Genes of 28 Significant Markers Identified through Localization of the 28 Shared SNP Markers.

Supplementary Table 5 Shared Marker Trait Associations (MTAs) among Traits.

Supplementary Table 6 Position and Distribution of all SNP Markers Identified by GWAS.

Supplementary Table 7 All markers associated with NB and their statistical significance (FDR).

Supplementary Table 8 All markers associated with NFW and their statistical significance (FDR).

Supplementary Table 9 All markers associated with NDW and their statistical significance (FDR).

Supplementary Table 10 All markers associated with DR and their statistical significance (FDR).
