# Supplementary material for: Genome-wide association study reveals SNP markers controlling drought tolerance and related agronomic traits in chickpea across multiple environments
Source: Front Plant Sci. 2024 Mar 8;15:1260690. doi: 10.3389/fpls.2024.1260690 (PMC10957531; doi:10.3389/fpls.2024.1260690)
Supplement: Supplementary file 1 [file DataSheet_1.pdf]

Chickpea supplementary Figures and Tables

December 24, 2023

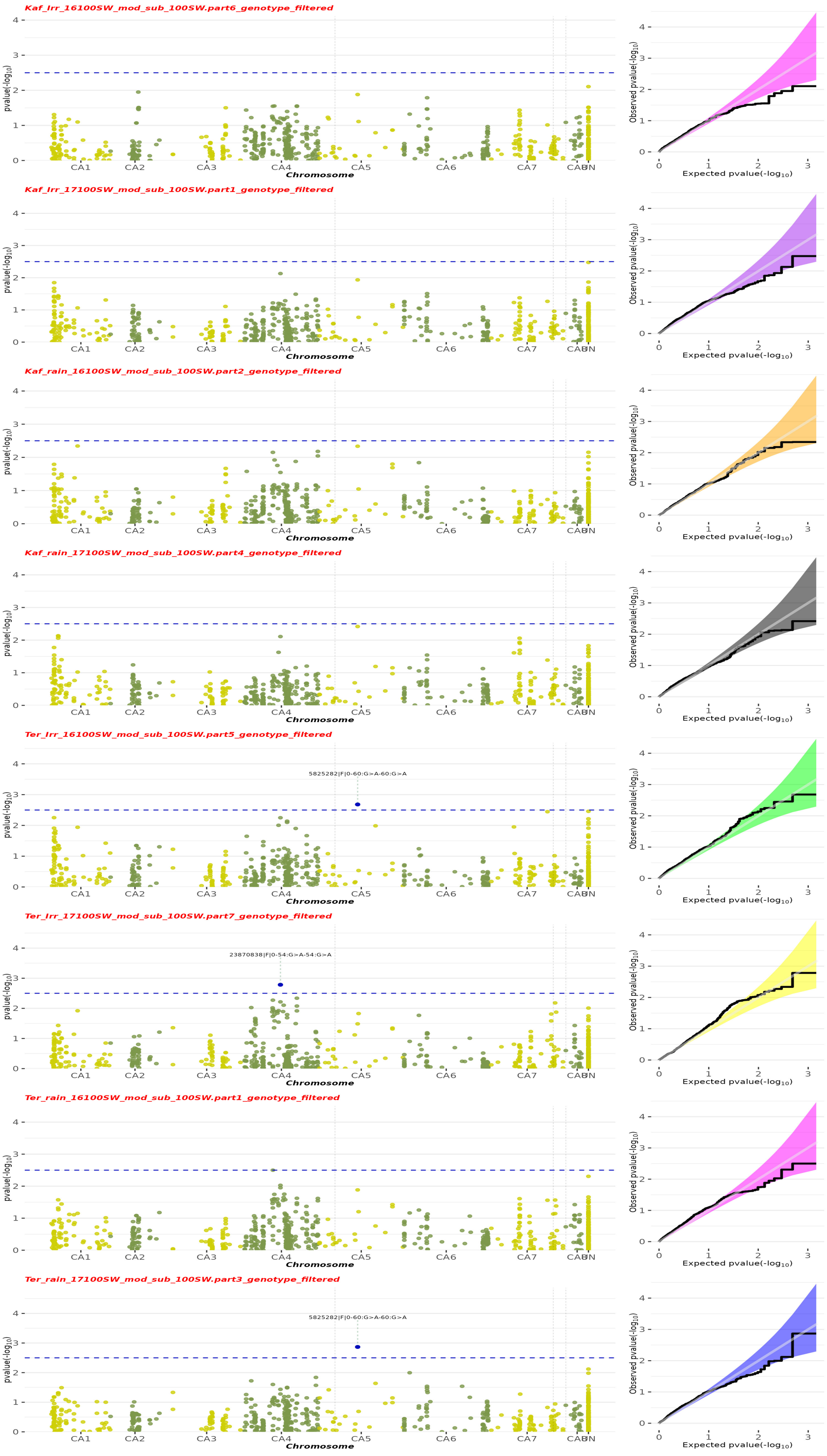

Figure 1: Manhattan plot for 100 seed weight trait

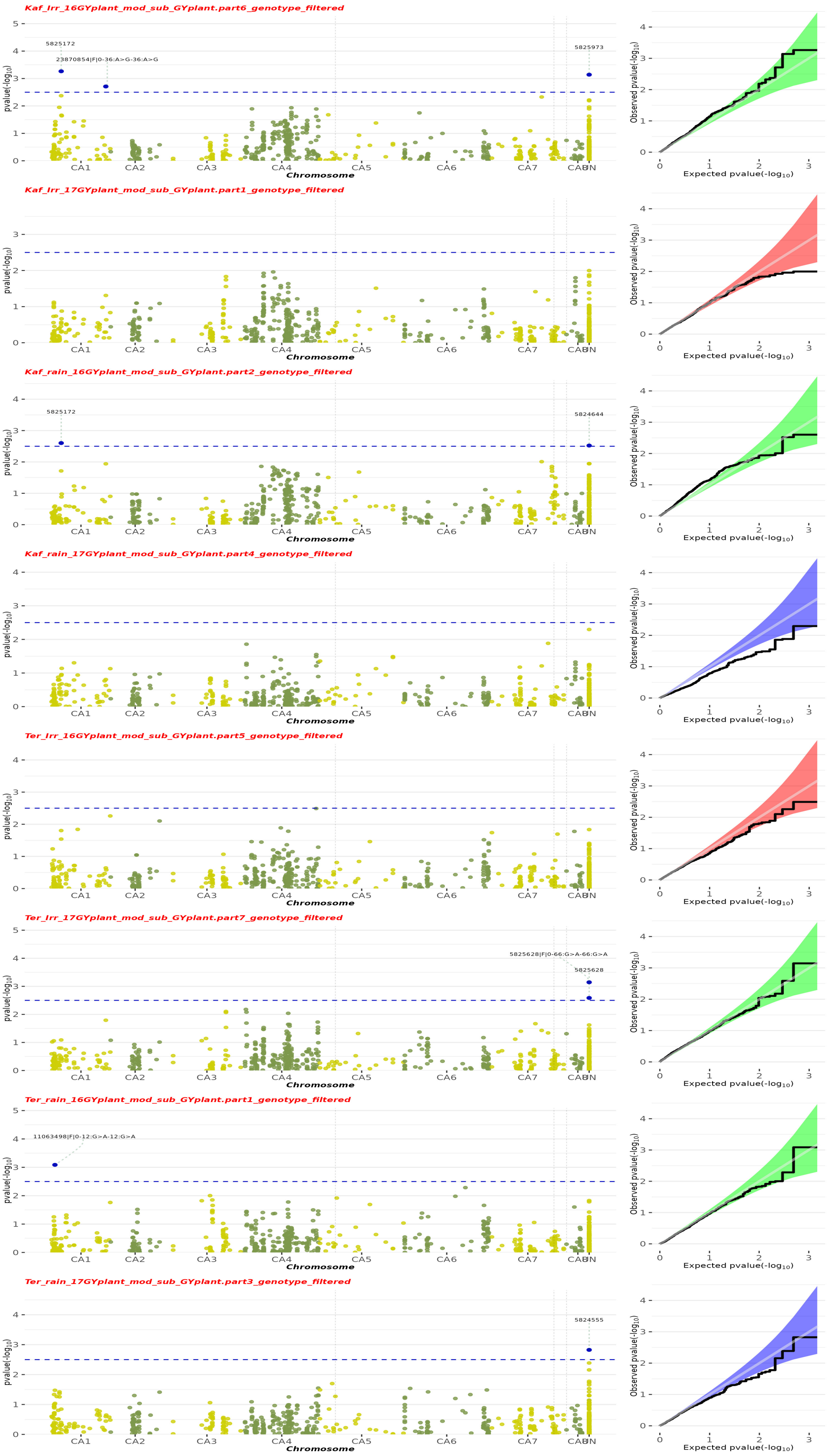

Figure 2: Manhattan plot for grain yield trait

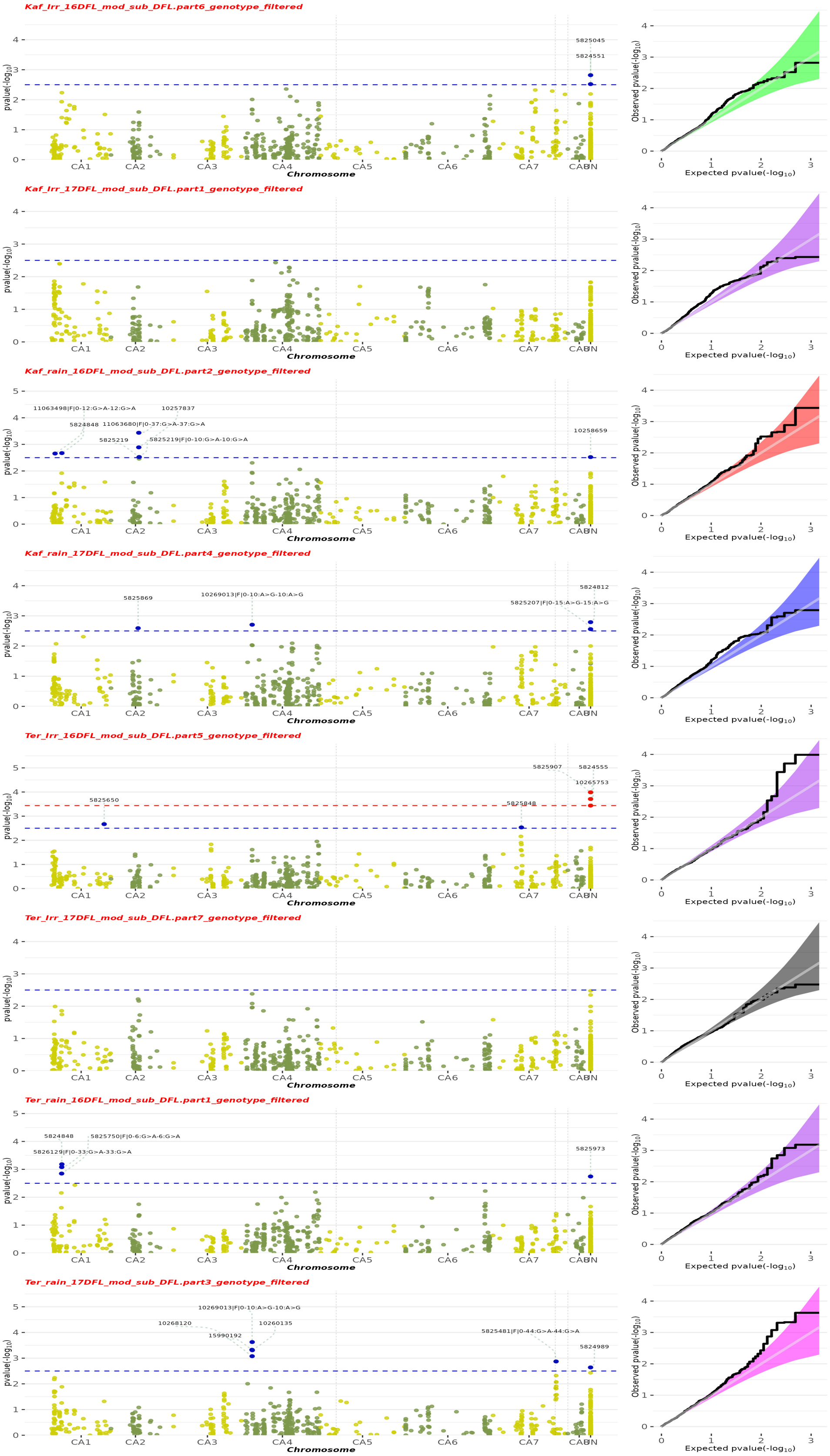

Figure 3: Manhattan plot for days to 50 flowering trait

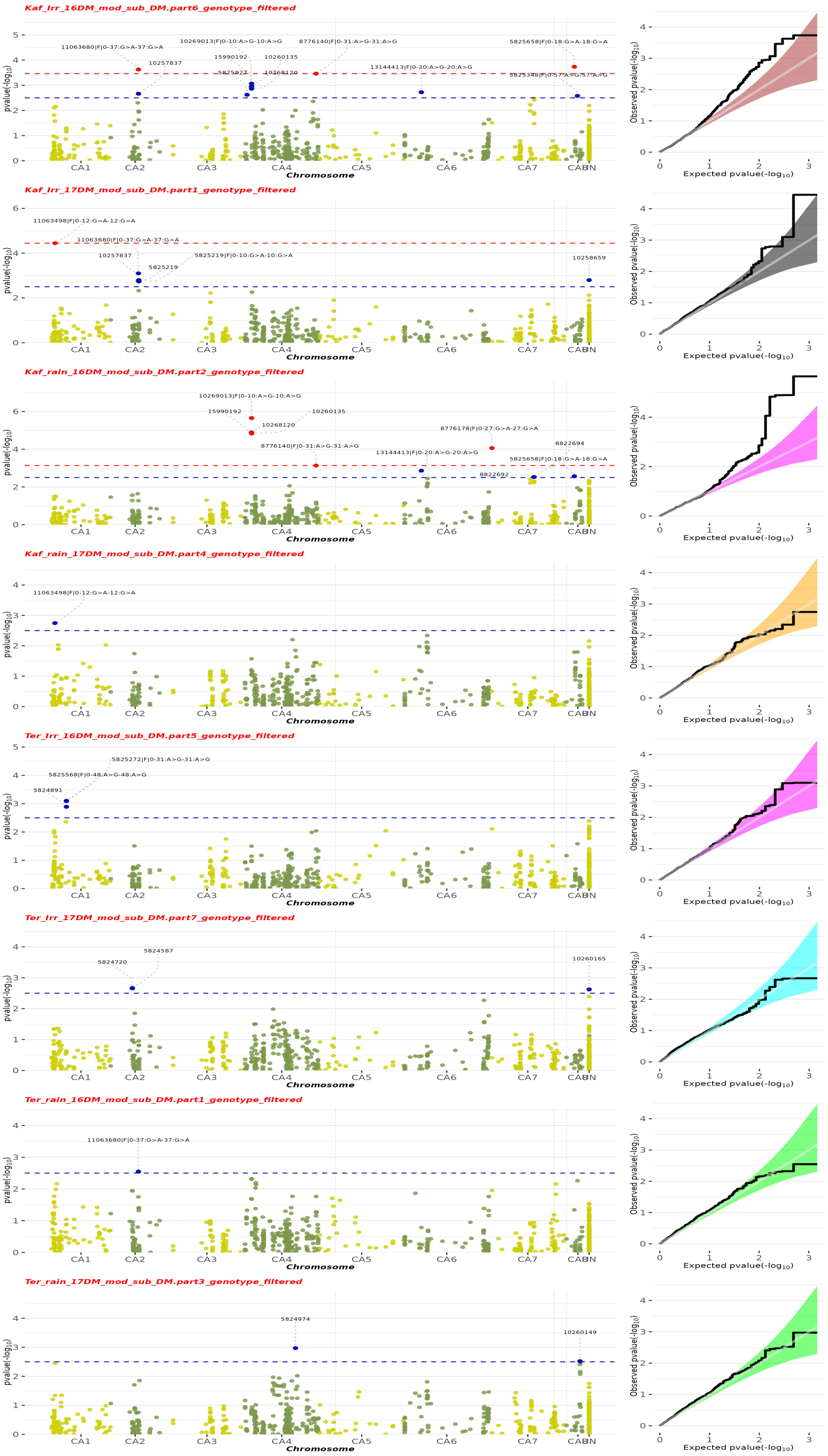

Figure 4: Manhattan plot for days to maturity trait

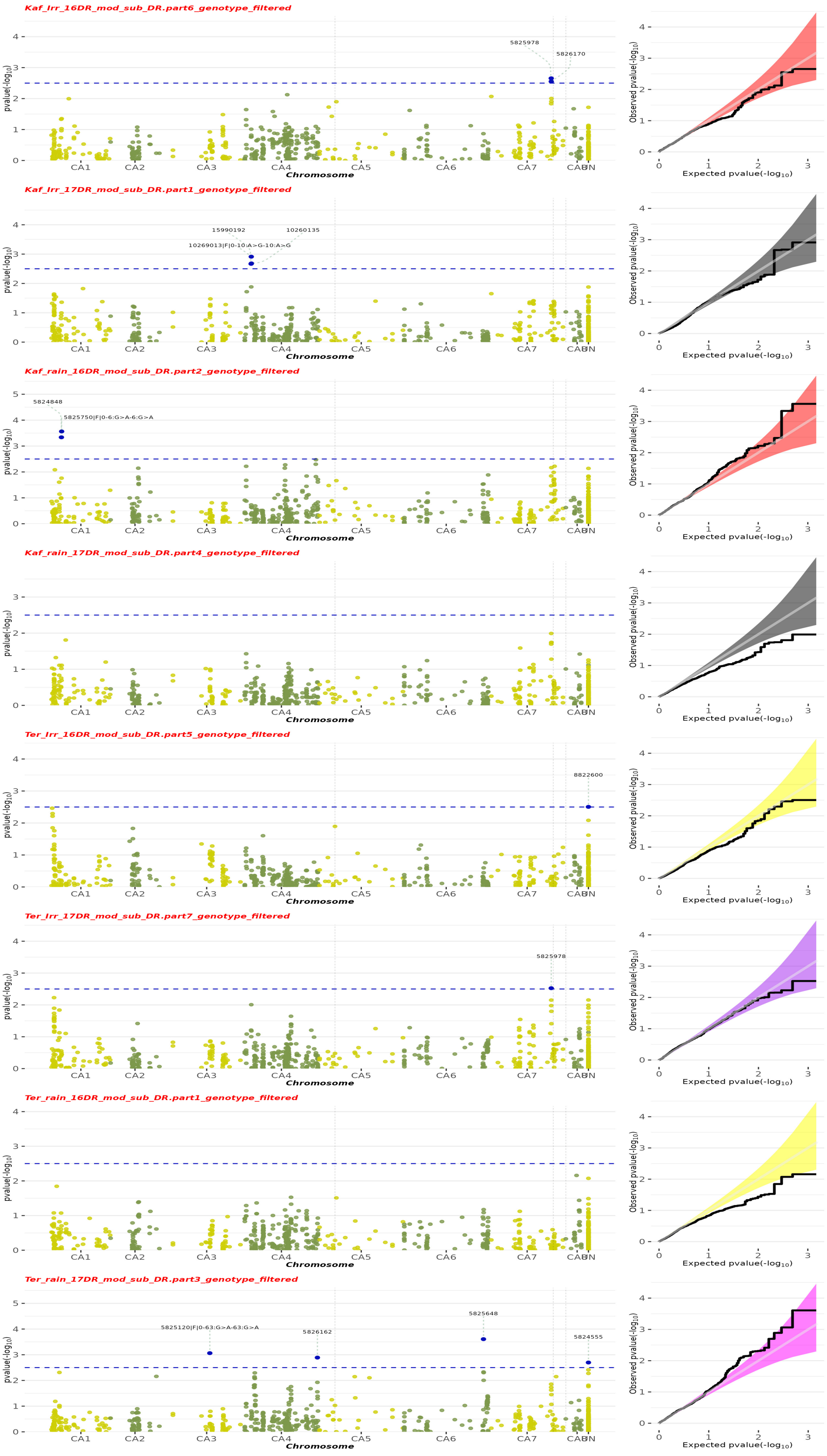

Figure 5: Manhattan plot for drought tolerance stress trait

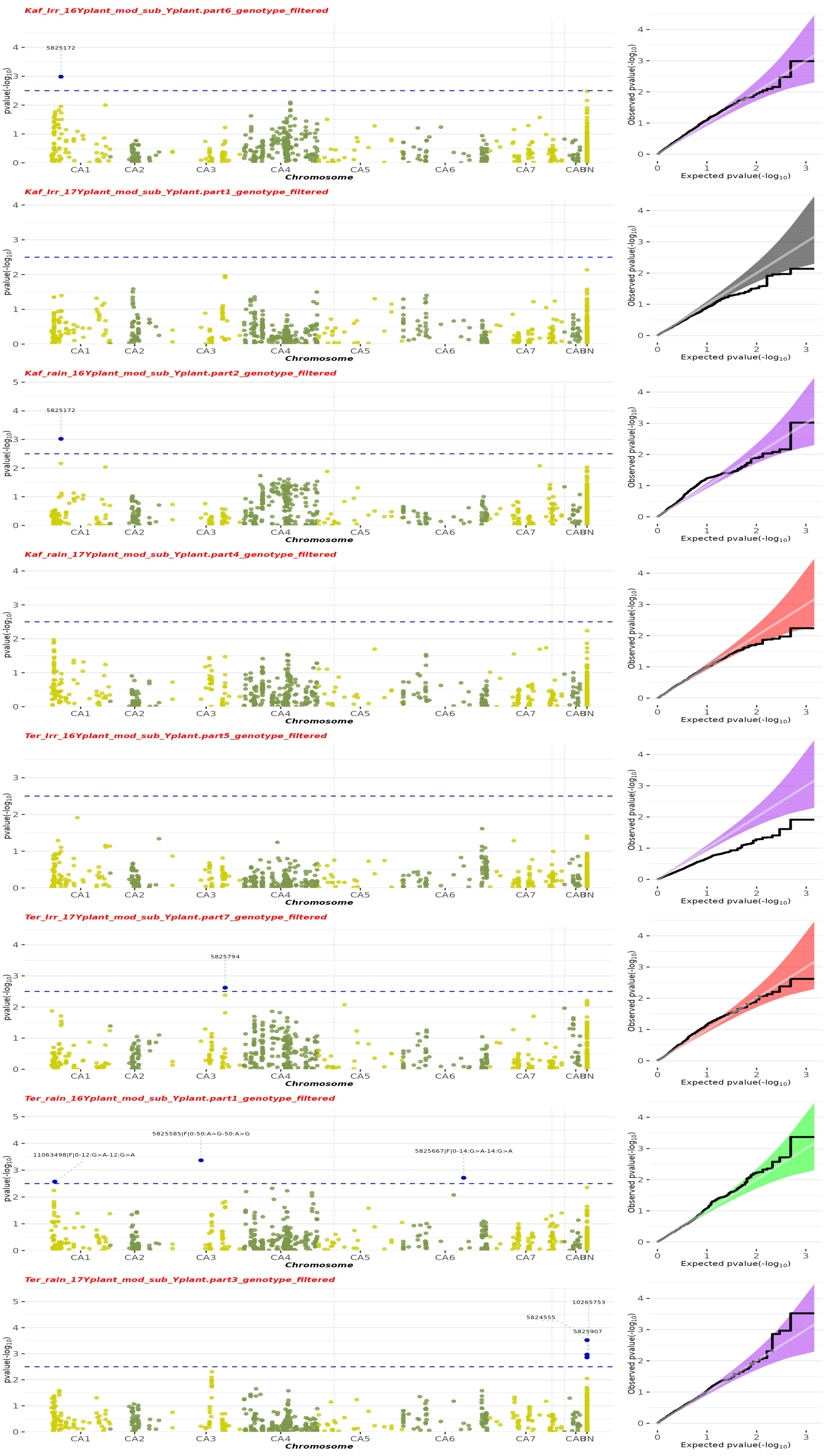

Figure 6: Manhattan plot for biological yield trait

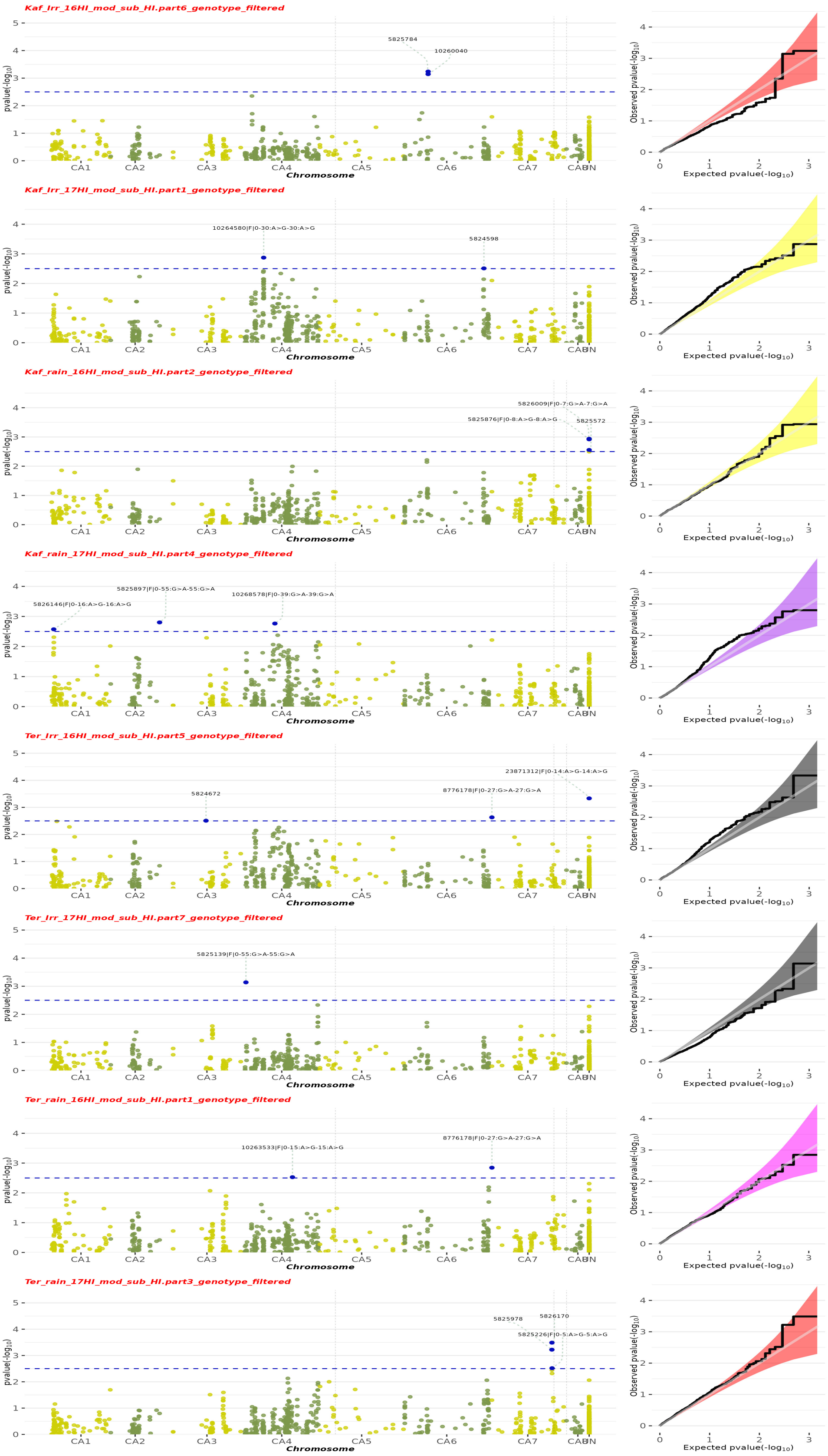

Figure 7: Manhattan plot for height index trait

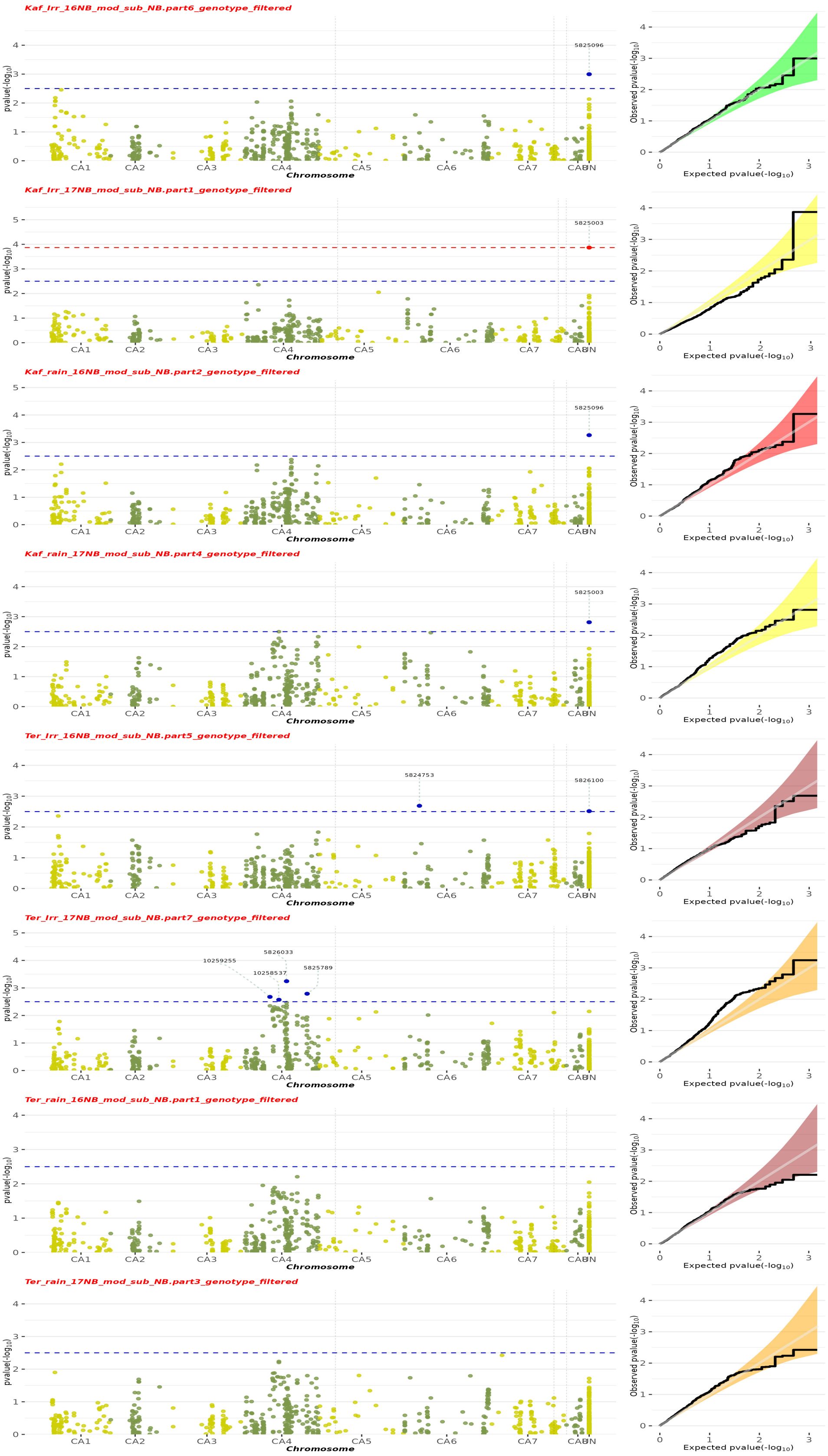

Figure 8: Manhattan plot for nodule biomass trait

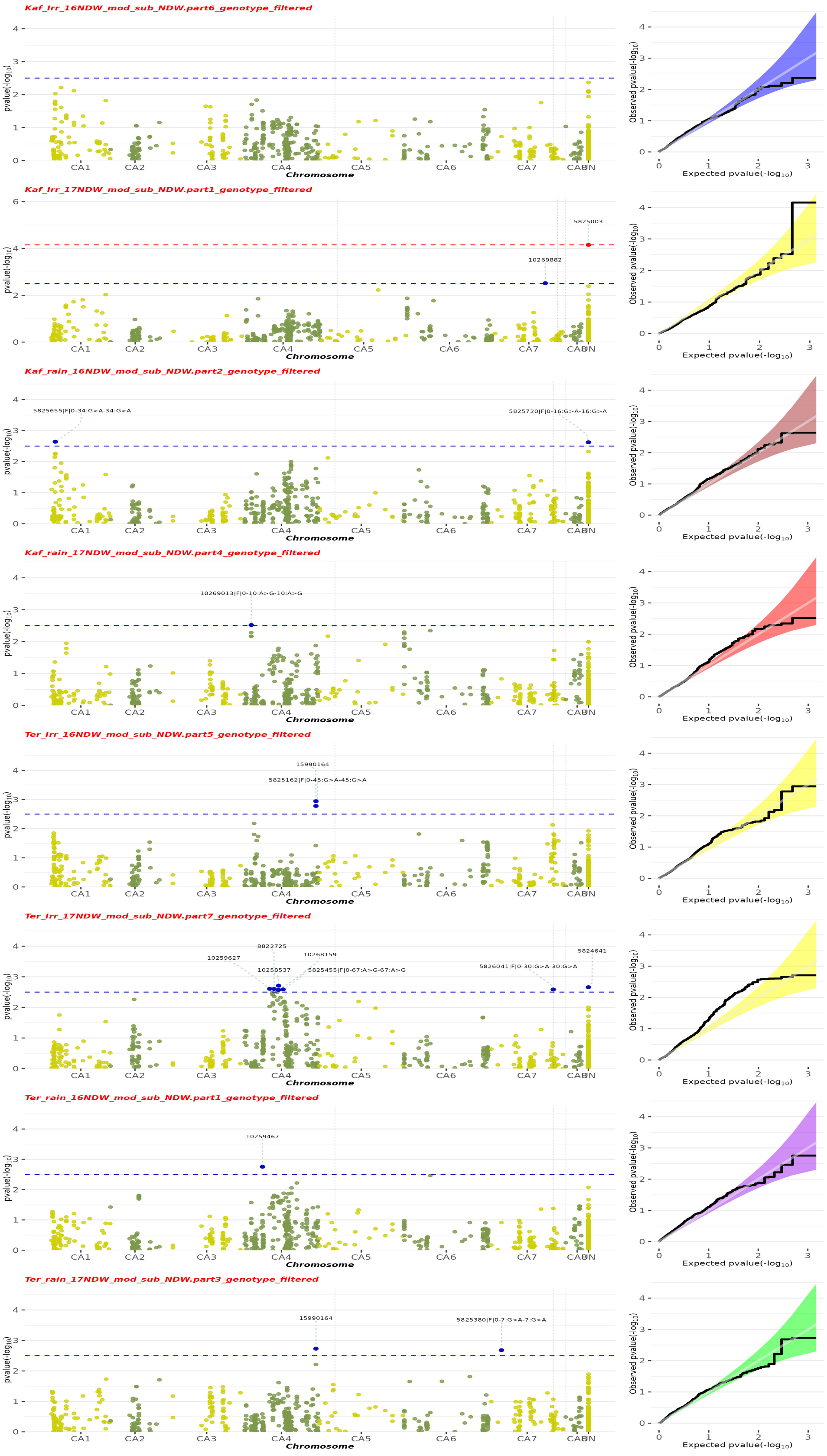

Figure 9: Manhattan plot for nodule dry weight trait

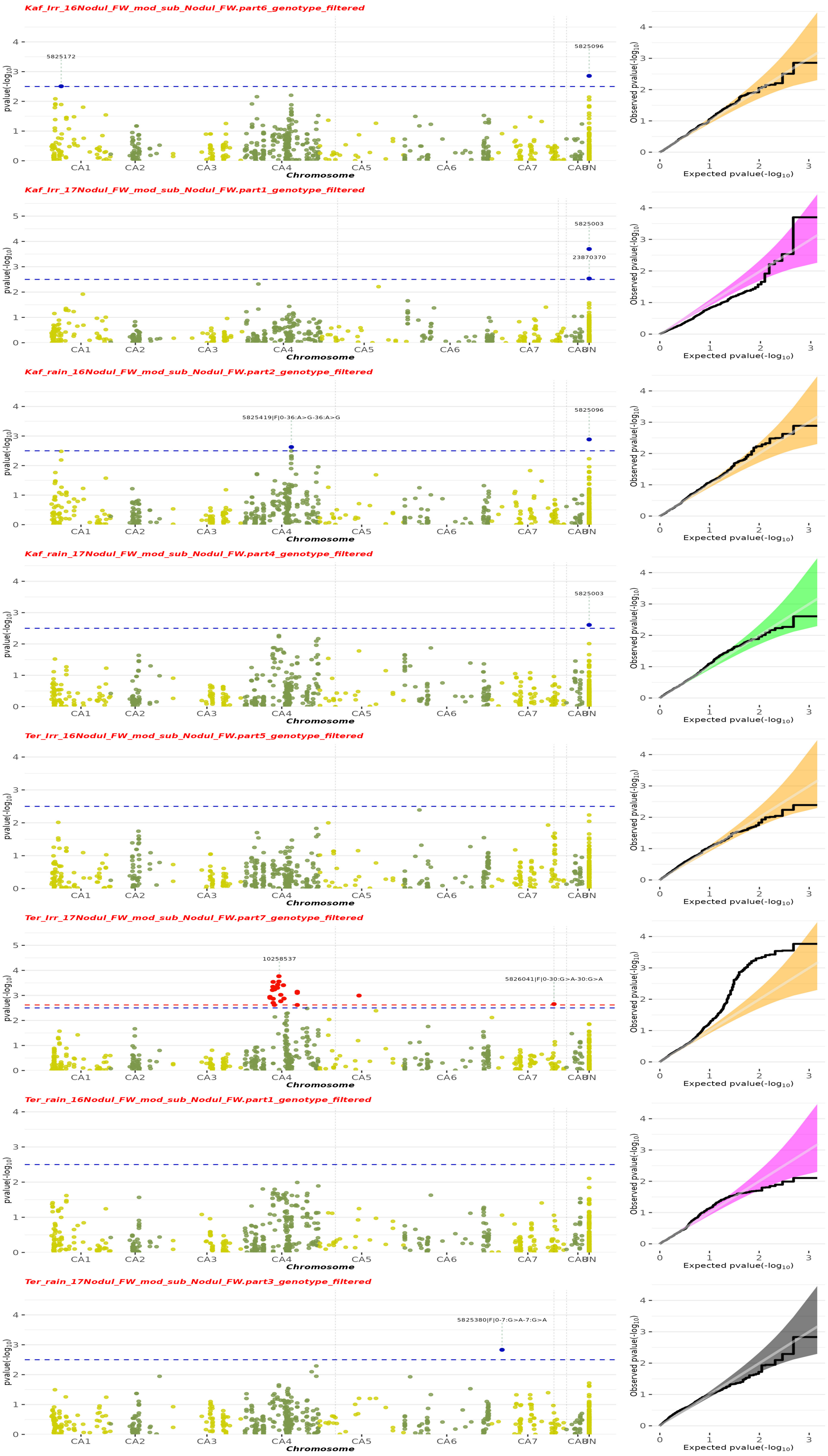

Figure 10: Manhattan plot for nodule fresh weight trait

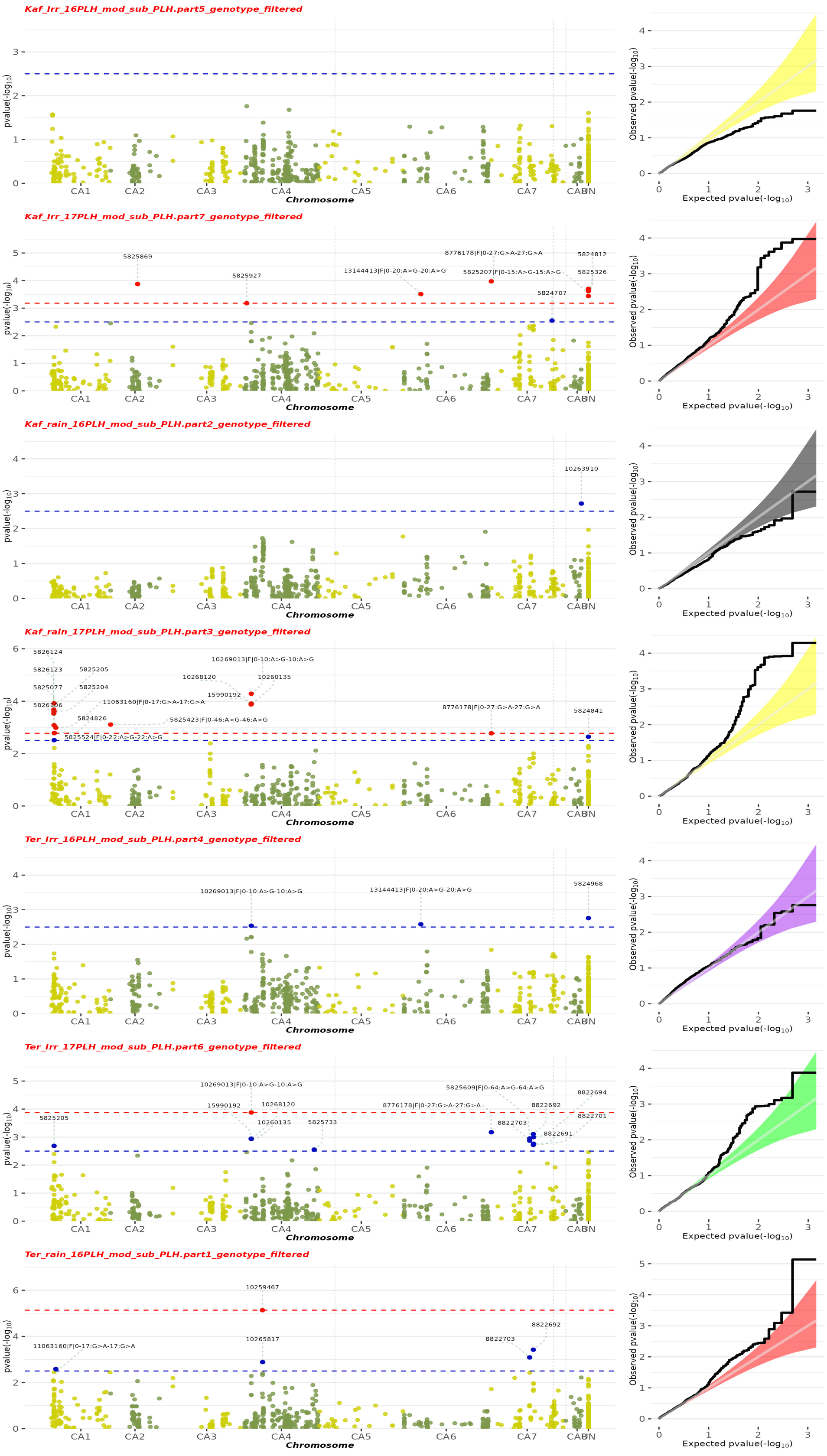

Figure 11: Manhattan plot for plant height trait
